# Axially Symmetric U−O−Ln‐ and U−O−U‐Containing Molecules from the Control of Uranyl Reduction with Simple f‐Block Halides

**DOI:** 10.1002/anie.201705197

**Published:** 2017-08-07

**Authors:** Polly L. Arnold, Bradley E. Cowie, Markéta Suvova, Markus Zegke, Nicola Magnani, Eric Colineau, Jean‐Christophe Griveau, Roberto Caciuffo, Jason B. Love

**Affiliations:** ^1^ EaStCHEM School of Chemistry University of Edinburgh The King's Buildings Edinburgh EH9 3FJ UK; ^2^ European Commission Directorate for Nuclear Safety and Security, Joint Research Centre Postfach 2340 76125 Karlsruhe Germany

**Keywords:** f-block chemistry, lanthanides, molecular magnetism, reduction, uranyl complexes

## Abstract

The reduction of U^VI^ uranyl halides or amides with simple Ln^II^ or U^III^ salts forms highly symmetric, linear, oxo‐bridged trinuclear U^V^/Ln^III^/U^V^, Ln^III^/U^IV^/Ln^III^, and U^IV^/U^IV^/U^IV^ complexes or linear Ln^III^/U^V^ polymers depending on the stoichiometry and solvent. The reactions can be tuned to give the products of one‐ or two‐electron uranyl reduction. The reactivity and magnetism of these compounds are discussed in the context of using a series of strongly oxo‐coupled homo‐ and heterometallic poly(f‐block) chains to better understand fundamental electronic structure in the f‐block.

The d^0^f^0^ uranyl ion, [UO_2_]^2+^, is the most common form exhibited by uranium in molecular complexes; it is a water‐soluble ion with linear, strongly bonded, and unreactive oxo groups.^1^ The reduction to [U^V^O_2_]^+^ and then insoluble U^IV^ by metals, minerals, or microbes is important for keeping it immobilized in the environment and out of groundwater, but the mechanisms for this are still not clear.^2^ The U^V^ uranyl ion can also provide better models for the highly radioactive and more oxo‐basic f^1^ and f^2^ neptunyl and plutonyl ions found in nuclear waste.^3^

Two seminal, coincident reports of the potassium‐reduced coordination polymer {[UO_2_(py)_5_][KI_2_(py)_2_]}_∞_ (**A**) showed that anaerobic uranyl(V) complexes are stable against disproportionation;^4^ since then, significant advances have been made in isolating uranyl(V) complexes. The reductive oxo functionalization with Group 1,^5^ Group 2,1c d‐block,1c, 6 rare‐earth,6b, 7 actinide,3a and main‐group metals5c, 8, 9 as well as silicon1d, 5b, 10 is also possible. The linearity of the f^1^ uranyl also provides a rare opportunity in f‐block chemistry to control the orientation of the magnetic vector, and uranyl(V)/transition‐metal single‐molecule magnets have been made through this oxo‐bridging strategy.3a, 6a, 7, 11 Lanthanide/actinide complexes are promising candidates for single‐molecule magnetism,11a and it has been proposed that an even higher degree of axial symmetry should produce exceptional molecular magnets.^12^

However, access to this new chemistry has almost exclusively been achieved through deployment of complicated polydentate ligands to saturate the equatorial coordination sphere of the uranyl ion.10c The only examples of oxo functionalization of simple systems involve the treatment of uranyl dichloride with a Group 1 reagent, namely a reduction with either K(Hg) or K[C_5_R_5_] (R=H, Me) to make **A** 4b and a reduction with NaCH_2_SiMe_3_ (followed by quenching with Me_3_SiCl) to make [U^IV^(OSiMe_3_)_2_I_2_(OPPh_3_)_2_] (**B**).^13^

Herein, we report how simple low‐oxidation‐state lanthanide and actinide salts can be used to oxo‐coordinate and reduce simple uranyl(VI) salts to make new classes of stable, highly symmetric, 4f and 5f ion oxo‐bridged uranyl(V) and uranium(IV) complexes.

The uranyl(VI) complex [UO_2_Cl_2_(THF)_2_] reacts with 1.25 equiv of a strongly reducing Ln^II^ halide, exemplified here by [SmI_2_(THF)_2_] or DyI_2_, in pyridine to form the reduced uranyl U^V^/Ln^III^/U^V^ complexes [{UO_2_(py)_5_}_2_(LnI_4_)]I (Ln=Sm (**1‐Sm**, 40 %), Dy (**1‐Dy**, 67 %); Scheme 1 a). The two (not isolated) byproducts are 0.5 equiv of LnCl_3_ from the redox reaction and 0.25 equiv of LnCl_2_, which results from a halide exchange that ensures that the uranyl products contain only iodide anions. Reactions carried out with 1 equiv of LnI_2_ and additional iodide in the form of ^n^Bu_4_NI also make **1** but less cleanly (see the Supporting Information). The formal oxidation states were confirmed by elemental analysis, FTIR spectroscopy, and single‐crystal X‐ray diffraction (Figure 1 a for **1‐Sm** and the Supporting Information for **1‐Dy**). In agreement with the assigned formal oxidation states, an asymmetric [OUO] stretching frequency is located at 818 (**1‐Sm**) and 825 cm^−1^ (**1‐Dy**; Nujol). This indicates stronger U=O overlap in these monometalated [O=U^V^=O]^+^ units than in dipotassiated [O=U^V^=O]^+^ in **A** (*ν*_OUO_=797 cm^−1^, KBr)4a although they are close to the those of the dilithiated [{UO_2_(py)_5_}{Li_2_(OTf)_3_}]_∞_ (*ν*_OUO_=816 cm^−1^; pyridine) and [UO_2_(OTf)(THF)_*n*_] (*ν*_OUO_=812 cm^−1^; pyridine) systems.^14^

**Figure 1:**
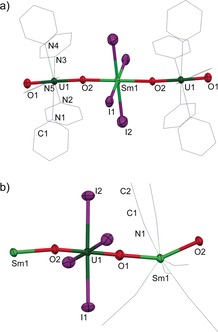
a) Solid‐state structure of the cation of **1‐Sm**⋅3 py with ellipsoids drawn at 50 % probability and coordinated solvent in wireframe.^19^ For clarity, hydrogen atoms, lattice solvent molecules, and iodide counteranions are omitted. Selected distances [Å] and angles [°]: mean U–O 1.859, Sm–O 2.331, Sm–I 3.053; O‐U‐O 177.81(2); U–N(py) 2.575(7)–2.623(8), Sm–I 2.993(1)–3.1137(6). b) Solid‐state structure of **2‐Sm** with ellipsoids drawn at 50 % probability and coordinated solvent in wireframe. For clarity, hydrogen atoms are omitted. Selected distances [Å] and angles [°]: mean U–O 1.875, U–I 3.11, Sm–O 2.334, Sm–N 2.537; O‐U‐O 179.3(2), O‐Sm‐O 145.6(2).

**Scheme 1:**
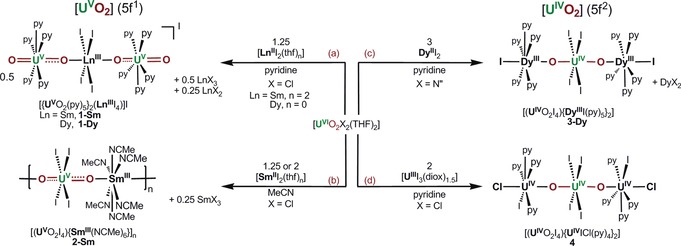
Synthesis of mixed lanthanide–actinide complexes by one‐electron reduction (left‐hand side) and by two‐electron reduction (right‐hand side) of simple uranyl salts to afford the singly reduced uranyl complexes a) [{UO_2_(py)_5_)}_2_(LnI_4_)]I (**1‐Sm** and **1‐Dy**) and b) [(UO_2_I_4_){Sm(NCMe)_6_}]_*n*_ (**2‐Sm**) as well as the doubly reduced uranyl salts c) [(UO_2_I_4_){DyI(py)_5_}_2_] (**3‐Dy**) and d) [(UO_2_I_4_){UICl(py)_4_}_2_] (**4**). N′′=N(SiMe_3_)_2_, diox=dioxane.

When the U^VI^/Sm^II^ reaction was repeated in acetonitrile, the polymeric analogue [(UO_2_I_4_)_2_{Sm(NCMe)_6_}]_*n*_ (**2‐Sm**) was isolated (Scheme 1 b). Elemental analysis repeatedly gives a formula of [(UO_2_I_4_)_2_{Sm(NCMe)_5_}]_*n*_, even for samples dried at ambient pressure. The solid‐state structure (Figure 1 b) shows that the iodide ligands now bind equatorially to the uranyl moiety while the solvent molecules bind to the harder Sm cation; this is in contrast to the structure of **1**, but in accordance with the HSAB principle.

Once formed, polymeric **2‐Sm** is no longer soluble, but FTIR spectra of the solid show that both oxo groups of the uranyl are now Ln‐coordinated as the uranyl stretch is significantly weakened to 722 cm^−1^. Unfortunately, the stronger reductant DyI_2_ reacts with MeCN,^15^ so we were unable to target **2‐Dy**.

Reactions between [UO_2_X_2_(THF)_2_] and two equivalents of strongly reducing Dy^II^ or U^III^ formed linear M−O−U^IV^−O−M oxo‐bridged trimers, such as [(UO_2_I_4_){UICl(py)_4_}_2_] (**4**; Scheme 1 d, M=U^IV^, X=Cl). Additionally, simple uranyl compounds other than the dichloride can be used; for example, [UO_2_{N(SiMe_3_)_2_}_2_(THF)_2_] reacted with two Dy^II^ ions to form the linear, symmetric Dy^III^/U^IV^/Dy^III^ complex [(UO_2_I_4_){DyI(py)_5_}_2_] (**3‐Dy**; see Figures S14–S16 for IR and Raman spectra).

When dissolved in pyridine, both canary‐yellow **1‐Sm** and cherry‐red **1‐Dy** gave cherry‐red solutions. The UV/Vis/NIR spectra exhibit strong, broadened absorptions with maxima at 461 nm (**1‐Sm**, *ϵ*=2140 m ^−1^ cm^−1^) and 454 nm (**1‐Dy**, *ϵ*=1249 m ^−1^ cm^−1^), which were assigned to π–π* processes. Additional f–f transitions were also observed between 650 and 1500 nm for complexes **1‐Sm** (*ϵ*=7–32 m ^−1^ cm^−1^) and **1‐Dy** (*ϵ*=13–34 m ^−1^ cm^−1^). Unfortunately, once precipitated from solution, **2‐Sm, 4**, and **5‐Sm** (see below) have very low solubility in organic solvents, preventing NMR or quantitative UV/Vis/NIR spectroscopic investigations (see the Supporting Information for solid‐state spectra).

Preliminary studies showed that the equatorial ligands can be exchanged. For example, the addition of 18‐crown‐6 to the reaction mixture of [UO_2_Cl_2_(THF)_2_] and [SmI_2_(THF)_2_] in THF afforded the crown ether solvated U^V^/Ln^III^/U^V^ complex [{UO_2_(18‐c‐6)}_2_(SmI_4_)]I (**5‐Sm**; Scheme 2; see the Supporting Information for characterization data). Alternatively, the pyridine ligands of **1‐Sm** can be substituted by 18‐crown‐6, underlining the stability of the U−O−Ln−O−U core.

**Scheme 2:**
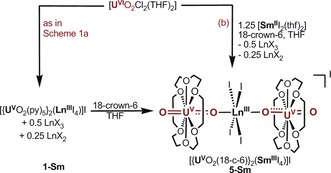
Reactions to afford the singly reduced uranyl complex [{UO_2_(18‐c‐6)}_2_(LnI_4_)]I (**5‐Sm**), either by the incorporation of 18‐crown‐6 in the reaction to form **1‐Sm** or from the reaction of **1‐Sm**.

Complexes **1** are isostructural, with a linear, pseudo‐*D*_2*h*_ [OUO]‐Ln‐[OUO] geometry (Figure 1). The uranium centers possess pentagonal‐bipyramidal geometry, retaining a *trans* di‐oxo coordination (O‐U‐O 177.8(3), U‐O‐Ln1 176.1(3), and O‐Ln1‐O 180.00(2)° in **1‐Sm**). In agreement with the formal U^V^ oxidation state, both the terminal U−O and Ln‐bridged U−O bonds are elongated with respect to uranyl dichloride, with lengths of 1.802(6) and 1.915(6) Å, respectively, in **1‐Sm**. The Sm−O and Dy−O bond lengths in **1** are consistent with a single bond (2.331(6) Å in **1‐Sm**), but longer than those in our previously reported [U^V^O_2_]^+^ *endo*‐oxo Ln‐metalated complex [{UO_2_Ln(py)_2_(L)}_2_] **C** (Sm–O 2.234(2) Å; L=Pacman‐shaped pyrrole macrocycle)6d, 7 and *exo*‐oxo M‐metalated complexes [{(X_*n*_M‐O)UO(THF)}(H_2_L)] (M=Li, K, Mg, Al, Zn, U, Np).1c, 3a, 9, 15 The average U−O bond length of 1.859 Å in **1‐Sm** is longer than that of 1.838 Å in the coordination polymer {[UO_2_(py)_5_][KI_2_(py)_2_]}_∞_ (**A**).^4^

The mean U^V^=O and Sm−O bond lengths in **2‐Sm** (Figure 1 b) are 1.876 Å and 2.335 Å, respectively, in accordance with the assigned U^V^/Sm^III^ formal oxidation states. The OUO unit remains essentially linear (O‐U‐O 179.3(2)°) but overall the metal oxo chain waves owing to a distortion of the O‐Sm‐O bond angle to 145.6(2)° as six coordinated MeCN molecules cannot fit in a purely equatorial plane.

The X‐ray structures of **3‐Dy** and **4** confirmed that the double reduction and oxo coordination remove any “uranyl ion” character, but form strong M−O−M′ interactions. We propose that the preferential coordination of iodide instead of pyridine to the central U^IV^ ion is a further indication of the loss of uranyl character as the use of the majority of valence orbitals in forming U=O bonds is a characteristic actinyl ion bonding feature. The binding of Cl *trans* to the oxo unit in **4** is ascribed to its stronger inverse *trans* influence (ITI) compared to that of iodide.^16^

The structures of **3‐Dy**⋅4 py and **4**⋅4 py have pseudo‐*D*_2*h*_ or *C*_2*h*_ symmetry, formally comprising [U^IV^O_2_I_4_]^2−^ and two pseudo‐pentagonal‐bipyramidal [Dy^III^I(py)_5_] or [U^IV^ICl(py)_4_]^+^ caps, respectively, with O‐U‐O=177.7(1)° in **3‐Dy** and O‐U‐O=180.0° in **4** (Figure 2). The U−O bonds in **3‐Dy** are significantly shorter than those found for the central [UO_2_I_4_]^2−^ unit in **4** (2.058(3) and 2.068(3) Å vs. 2.166(5) Å), whereas the U−I bonds in **3‐Dy** are significantly longer than those in **4** (3.1425(4)–3.1618(4) Å vs. 3.0436(8)–3.0572(8) Å). Furthermore, the Dy−O bonds in **3‐Dy** (2.126(3) and 2.119(3) Å) are significantly shorter than those in **1‐Dy** (2.270(5) Å), which is consistent with reduction of U^V^ to U^IV^ and a more symmetric oxo bridging in **3‐Dy**. In **4**, the outer U1−O1 bond is much shorter than the central U2−O1 bond (2.042(5) vs. 2.166(5) Å), but both are shorter than the average U−O bond in the CSD (2.361 Å).

**Figure 2:**
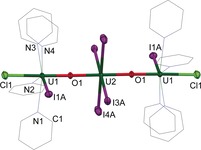
Solid‐state structure of **4**⋅4 py with ellipsoids drawn at 50 % probability.^19^ For clarity, hydrogen atoms, lattice solvent molecules, and lower‐occupancy positions of the disordered I and N_3_‐py are omitted. Selected distances [Å] and angles [°]: mean U–O 2.058(3), U2–I3 3.0571, U1–I1 3.0678(7); U‐O‐U 173.7, O‐U‐O 180.0.

Anticipating that the linear geometry of these complexes could generate interesting magnetic behavior, the dc magnetic susceptibilities *χ* of **1‐Sm** and **4** were recorded as a function of the temperature *T* (Figure 3).

**Figure 3:**
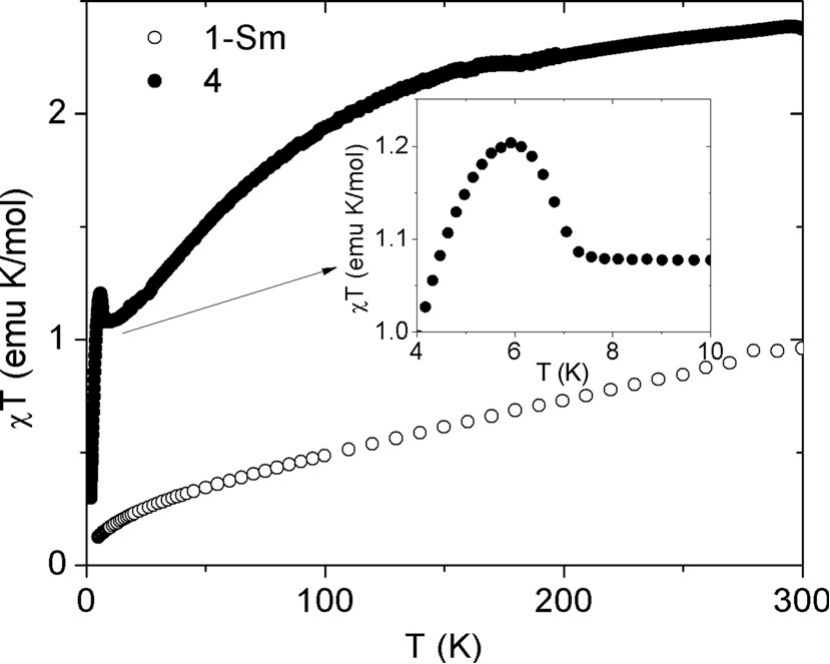
The dc magnetic susceptibility χ as a function of temperature (plotted as *χT* vs. *T*) of **1‐Sm** (○) and **4** (•). The inset shows the increase in *χT* with decreasing temperature between 8 and 6 K.

The susceptibility is significantly larger for **4** than for **1‐Sm** over the whole investigated temperature range, a fact that is at first counterintuitive given that the latter complex is made up of three Kramers ions whereas the former contains three non‐Kramers ions. This is explained by considering that the free‐ion effective magnetic moment *μ*_eff_ of U^IV^ is much larger than those of both U^V^ and Sm^III^, and that the high‐symmetry, linear geometry of **4** cannot completely remove the degeneracy of the low‐energy ligand‐field levels (and therefore cannot isolate a non‐magnetic singlet as the ground state). Furthermore, whereas the susceptibility for **1‐Sm** is essentially featureless, the *χT* versus *T* plot for **4** shows a clear upturn between 8 and 6 K followed by a sharp drop upon further decreasing the temperature. Such an upturn is usually considered as a signature of ferromagnetic coupling, but it is more likely that each of the two outer U^IV^ ions interacts antiferromagnetically with the central one, whilst also carrying a larger magnetic moment. Indeed, fitting the high‐temperature part of the inverse susceptibility of **4** (Figure S6) to the Curie–Weiss law *χ* ^−1^=8(*T*−*Θ*)/*μ*_eff_^2^ gives *μ*_eff_^2^=21.4 μ_B_^2^ and a negative Curie–Weiss temperature of *Θ*=−36.2 K, which indicates the presence of antiferromagnetic interactions. The effective magnetic moment is only slightly smaller than that for two U^IV^ ions and much lower than for three such ions. A similar situation was found in the trinuclear Np^VI/V^ neptunyl complex [(Np^VI^O_2_Cl_2_){Np^V^O_2_Cl(THF)_3_}_2_],^17^ including the sharp downturn in *χT* at very low temperature, which was attributed to magnetic saturation. As this Np complex is also a single‐molecule magnet with a high energy barrier to magnetic relaxation (about 100 cm^−1^), we investigated the ac susceptibility of **4**, but did not find any significant slowing down of the magnetic relaxation processes (Figure S7).

An a priori prediction of which Ln or An salts would be capable of one‐ or two‐electron uranyl reduction is complicated by the presence of strongly coordinating solvents, additional halides, as well as the known propensity of these oxophilic metals to rapidly change coordination geometry, number, and their Lewis acidity. For example, the coordination of Lewis acidic Li^+^, boranes, or 4f cations to the U^VI^ uranyl oxo group shifts the U^VI^ reduction by as much as +600 mV,5a, 10d and uranyl iodides are easier to reduce than their chloride analogues; UO_2_I_2_(py)_3_ can be reduced by the organic anions of the normally stable organometallic U(η‐C_8_H_8_)_2_ to form a hexa‐uranium cluster U_6_O_8_I_8_(py)_10_.^18^

In conclusion, we have demonstrated that redox reactions between readily accessible lanthanide and actinyl halides and related simple salts can produce linear oxo‐coupled 4f/5f oligomers or infinite chains. These can be tuned for one‐ or two‐electron reduction, and mono‐ or dioxo‐functionalization. The reductants can be 4f (exemplified by Sm^II^ and Dy^II^) or 5f cations (exemplified by U^III^). Some postsynthetic modifications have been demonstrated through simple L donor ligand exchange. The complexes are thermally stable, and most can be dissolved in a variety of aprotic solvents. Given their inherent symmetry and simplicity, these complexes may be an interesting and flexible system from which spectroscopic studies can elucidate more of the fundamental electronic structural details that are still missing for the f‐block, in particular for the f^1^ actinyl ions; preliminary magnetic analyses show strong antiferromagnetic coupling in the all‐U system. Work is in progress to extrapolate these methods to the transuranic neptunyl and plutonyl cations, and to other combinations to explore the electronic structures of these systems.

## Conflict of interest

The authors declare no conflict of interest.

## Supporting information

As a service to our authors and readers, this journal provides supporting information supplied by the authors. Such materials are peer reviewed and may be re‐organized for online delivery, but are not copy‐edited or typeset. Technical support issues arising from supporting information (other than missing files) should be addressed to the authors.

Supplementary
