# Supplementary material for: Axially Symmetric U−O−Ln‐ and U−O−U‐Containing Molecules from the Control of Uranyl Reduction with Simple f‐Block Halides
Source: Angew Chem Int Ed Engl. 2017 Aug 7;56(36):10775–9. doi: 10.1002/anie.201705197 (PMC5697649; doi:10.1002/anie.201705197)
Supplement: Supplementary file 1 — Supplementary [file ANIE-56-10775-s001.pdf]

Angewandte Chemie
Eine Zeitschrift der Gesellschaft Deutscher Chemiker

Supporting Information

Axially Symmetric U−O−Ln- and U−O−U-Containing Molecules from the Control of Uranyl Reduction with Simple f-Block Halides

Polly L. Arnold, Bradley E. Cowie, Markéta Suvova, Markus Zegke, Nicola Magnani, Eric Colineau, Jean-Christophe Griveau, Roberto Caciuffo,* and Jason B. Love**

anie_201705197_sm_miscellaneous_information.pdf

Author Contributions

P.A. Conceptualization: Equal; Data curation: Lead; Formal analysis: Equal; Funding acquisition: Lead; Investigation: Equal; Methodology: Equal; Project administration: Equal; Supervision: Equal; Validation: Equal; Visualization: Lead; Writing—review & editing: Lead
B.C. Formal analysis: Equal; Investigation: Lead; Writing—original draft: Equal; Writing—review & editing: Supporting
M.S. Formal analysis: Supporting; Investigation: Supporting
M.Z. Investigation: Supporting
N.M. Formal analysis: Supporting; Writing—original draft: Supporting
E.C. Formal analysis: Supporting
J.G. Investigation: Supporting
R.C. Formal analysis: Supporting; Funding acquisition: Supporting; Investigation: Supporting; Writing—original draft: Supporting
J.L. Conceptualization: Equal; Formal analysis: Equal; Investigation: Equal; Methodology: Equal; Project administration: Equal; Supervision: Equal; Validation: Equal; Writing—review & editing: Supporting.

Table of Contents

General Details 1

Syntheses 2

[{$UO_2(py)_5$}$_2$(SmI_4)]I (1-Sm) 2

[{$UO_2(py)_5$}$_2$(DyI_4)]I (1-Dy) 2

{[UO_2I_4][Sm(MeCN)$_6$]}$_n$ (2-Sm) 2

[(UO_2I_4){DyI(py)$_5$}$_2$]] (3-Dy) 3

[(UO_2I_4){UClI(py)$_4$}$_2$] (4) 3

Reactions of the multinuclear complexes 3

Reaction of 1-Sm with 18-crown-6; synthesis of [{UO_2(18-crown-6)}$_2$(SmI_4)]I (5-Sm) 3

Reaction of 1-Sm with KO^{t}Bu; synthesis of [{$UO_2(py)_5$}$_2$($KI_2(py)_2$)]$_n$ 4

Reaction of 1-Sm with KN($SiMe_3$)$_2$; synthesis of [{$UO_2(py)_5$}$_2$($KI_2(py)_2$)]$_n$ 4

General X-ray experimental details 4

Variable Temperature SQUID Magnetic Measurements and Analysis 11

FTIR and Raman Spectra 13

UVVisNIR Spectra 26

General Details

All manipulations were carried out under a dry, oxygen-free atmosphere of nitrogen using standard Schlenk and glovebox techniques. Pyridine and 1,4-dioxane were distilled from potassium and stored over 4 Å molecular sieves. Acetonitrile was distilled from CaH_2 and stored over 4 Å molecular sieves. THF, Hexanes and CH_2Cl_2 were sparged with N_2 then purified by passage through activated alumina and stored over 4 Å molecular sieves. All gases were supplied by BOC gases UK. All glassware items, cannulae and Fisherbrand 1.2 μm retention glass microfibre filters were dried at 150 °C overnight before use.

Infrared spectra were recorded on a Perkin Elmer Spectrum 65 FT-IR spectrometer as nujol mulls between NaCl disks. Raman spectra were recorded on a Renishaw InVia Raman spectrometer using either a Renishaw solid-state diode laser (excitation λ = 785 nm) or an argon ion gas discharge laser (excitation λ = 514 nm), each with a maximum power output of ~2 mW and laser diameter of ~3 μm. A Nikon L-plan objective microscope (×100 objective, NA 0.7) was used for sample focussing. Samples were prepared in Mettler ME-18552 melting point tubes plugged with Apiezon H-grease. The Raman spectra were measured in the range of 1600-100 cm^{-1} for the 785 nm wavelength laser or from 1700-100 cm^{-1} for the 514 nm wavelength laser. The laser power used for the 785 nm wavelength laser was either 50 % (focus = 100 %) with 15 scans of 45 seconds of exposure (compound **4**) or 100 % (focus = 100 %) with 15 scans of 25 seconds of exposure (**1-Sm**, **1-Dy**, **2-Sm**, **5-Sm**). The laser power used for the 514 nm laser was either 10 % (focus = 100 %) with 15 scans of 25 seconds of exposure (**1-Sm**), or 50 % (focus = 25 %) with 15 scans of 25 seconds of exposure (**2-Sm**, **3-Dy**, **4**, **5-Sm**). UV-vis-NIR spectra were recorded on a JASCO V-670 spectrophotometer using a sealed quartz cuvette fitted with a Young's tap. Pyridine solutions of **1-Sm** (0.44, 5.97 mM) and **1-Dy** (0.16, 4.7 mM), and CH_2Cl_2 solution of **1-Sm** (0.018, 0.16, 2.47 mM) and **1-Dy** (0.0196, 0.145, 2.86 mM) were prepared and analysed. Elemental analyses were carried out at London Metropolitan University, London, UK or Pascher Labor, Germany.

Single crystal X-ray diffraction data for **1-Sm·3py**, **1-Dy·3py**, **2-Sm** and **3-Dy·4py** were collected using an Excalibur Eos diffractometer, fitted with a CCD area detector and using MoKα

radiation (λ = 0.71073 Å). The data for **1-Sm·3py**, **1-Dy·3py** and **3-Dy·4py** were collected at 173(2) K, whereas those for **2-Sm** was collected at 298(2) K. Single crystal X-ray diffraction data for **4·2py** were collected using an Oxford Diffraction Supernova instrument at 120 K, fitted with a CCD area detector using MoKα radiation.

Iodine and $HN(SiMe_3)_2$ were purchased from Sigma-Aldrich and used as was. KO^tBu and 18-crown-6 were purchased from Sigma-Aldrich, the latter was dissolved in Et_2O then passed through molecular sieves (4 Å) before being dried *in vacuo*. KH (suspension in oil, Fischer Scientific) was washed with hexanes to remove the oil then dried *in vacuo*. $[UO_2Cl_2(THF)_2]$,[1] $KN(SiMe_3)_2$,[2] $[UO_2\{N(SiMe_3)_2\}_2(THF)_2]$,[3] $[SmI_2(THF)_2]$[4] and $[UI_3(dioxane)_{1.5}]$[5] were prepared according to literature procedures. DyI_2 was either purchased from Sigma-Aldrich and stored in the glove box, or prepared according to the literature procedure.[6]

Syntheses

$[\{UO_2(py)_5\}_2(SmI_4)]I$ (1-Sm)

a) Pyridine (50 mL) was added to a 200 mL Schenk flask containing $[UO_2Cl_2(THF)_2]$ (2.28 g, 4.69 mmol) and $[SmI_2(THF)_2]$ (3.24 g, 5.87 mmol), producing a deep red solution and a yellow precipitate. The reaction mixture was stirred for 2 hours at room temperature. After 48 hours standing at room temperature, the desired product, a lemon yellow solid, was isolated by filtration and dried under reduced pressure; yield = 1.80 g. A second batch was obtained from the filtrate upon concentration to half its volume and storage at –20 °C for a further two weeks; overall yield = 3.97 g (40 %). X-ray quality crystals were obtained via vapour diffusion of hexanes into a pyridine solution of **1-Sm** at room temperature. **Elemental Analysis Calcd (%)** for $C_{50}H_{50}I_5N_{10}O_4SmU_2$: C, 28.38; H, 2.38; N, 6.62. Found: C, 28.43; H, 2.49; N, 6.71 %. **IR:** ν(asym. UO_2^+) = 818 cm^{-1} (nujol). No X-band EPR resonances were discernible in four different samples of **1-Sm**.
b) $[UO_2Cl_2(THF)_2]$ (36.7 mg, 0.0757 mmol), $[SmI_2(THF)_2]$ (41.8 mg, 0.0757 mmol) and two equivalents of $[^nBu_4N]I$ (56.2 mg, 0.151 mmol) were combined and dissolved in 1 mL of pyridine, and allowed to stand at room temperature for 16h. The product mixture was evaporated to dryness under reduced pressure to yield a dark brown solid which was analysed by FTIR as a nujol mull, Figure S10 and 11, as containing a significant quantity of **1-Sm**, but also other products, so no further work-up was carried out.

$[\{UO_2(py)_5\}_2(DyI_4)]I$ (1-Dy)

a) In a vial, a pyridine (2 mL) solution of $[UO_2Cl_2(THF)_2]$ (40.0 mg, 8.25×10^{-2} mmol) and DyI_2 (86.0 mg, 0.205 mmol) was stirred at room temperature for 48 hours, producing a dark red solution. The reaction mixture was filtered then vapour diffusion of hexanes into the filtrate yielded **1-Dy·3py** as orange blocks suitable for X-ray diffraction. Yield = 65 mg (67 %). **Elemental Analysis Calcd (%)** for $C_{65}H_{65}DyI_5N_{13}O_4U_2$: C, 33.00; H, 2.77; N, 7.70. Found: C, 32.97; H, 2.69; N, 7.57 %. **IR:** ν(asym. UO_2^+) = 825 cm^{-1} (nujol).
b) A vial was charged with a pyridine solution of $[UO_2Cl_2(THF)_2]$ (20.5 mg, 0.0423 mmol, 1 mL), DyI_2 (35.5 mg, 0.0845 mmol) and $[^nBu_4N]I$ (15.7 mg, 0.0423 mmol) and allowed to stand at room temperature for 24 hours. The reaction mixture was then filtered to remove traces of insoluble material, and the filtrate was stored at room temperature in the presence of hexanes vapour. After several days, X-ray quality crystals of **1-Dy** (*ca* 5 mg, unoptimized) were obtained and their identity was confirmed by single crystal X-ray diffraction, so no further crystalline material was isolated.

$\{[UO_2I_4][Sm(MeCN)_6]\}_n$ (2-Sm)

Acetonitrile (50 mL) was added to a 100 mL Schlenk flask containing $[UO_2Cl_2(THF)_2]$ (0.536 g, 1.10 mmol) and $[SmI_2(THF)_2]$ (1.22 g, 2.21 mmol), resulting in the formation of a brown/green/yellow

solution. After approximately 1 hour the solution had lightened in colour and a yellow/green precipitate had formed; the reaction was allowed to stir overnight at room temperature. The solution was then filtered and the green/brown mother liquors were stored for 2 days at –20 °C, providing **2-Sm** as green/yellow needles which were isolated by filtration and dried under reduced pressure; yield = 0.861 g (66 %). X-ray quality crystals of **2-Sm** were obtained from storage of an acetonitrile solution of [UO_2Cl_2(THF)$_2$] and [SmI_2(THF)$_2$] at room temperature overnight. **Note:** Drying crystals of **2-Sm** under reduced pressure, or storing them in the absence of acetonitrile at ambient temperature and pressure N_2 atmosphere results in loss of one MeCN ligand. **Elemental Analysis Calcd (%)** for $C_{10}H_{15}I_4N_5O_2SmU$: C, 10.60; H, 1.33; N, 6.18. Found: C, 10.36; H, 1.43; N, 5.99. **IR:** ν(MeCN) = 2303, 2275 cm^{-1}; ν(asym. UO_2^+) = 722 cm^{-1} (nujol). Laser irradiation caused excessive fluorescence of the sample in the Raman experiment so the symmetric [UO_2]$^+$ stretch could not be located.

[(UO_2I_4){DyI(py)$_5$}$_2$]] (3-Dy)

Pyridine (1 mL) was added to a 3 mL vial containing [UO_2{N($SiMe_3$)$_2$}$_2$(THF)$_2$] (24.6 mg, 3.35×10^{-2} mmol) and DyI_2 (42.2 mg, 0.100 mmol), producing a red/orange coloured solution. The reaction solution was stored overnight at room temperature then filtered through glass filter paper to remove any insoluble material. The filtrate was then stored at room temperature for several days to obtain X-ray quality crystals of **3-Dy** via vapour diffusion with hexanes. Yield of single-crystal quality material (allowed to dry at room temperature inside the glove box under an N_2 atmosphere) 10 mg (14 %). **Elemental Analysis Calcd (%)** for $C_{50}H_{50}Dy_2I_6N_{10}O_2U$: C, 27.96; H, 2.35; N, 6.52. Found: C, 27.83; H, 2.43; N, 6.25 %.

[(UO_2I_4){UClI(py)$_4$}$_2$] (4)

Pyridine (10 mL) was added into 20 mL vial containing [UO_2Cl_2(THF)$_2$] (0.392 g, 0.808 mmol) and [UI_3(dioxane)$_{1.5}$] (1.22 g, 1.62 mmol) in the glove box, resulting in the formation of a deep red solution. After approximately 1 hour the solution had lightened in colour and a yellow/green precipitate had formed; the reaction was allowed to stir overnight at room temperature. The mother liquors were then decanted from the precipitated product, and the remaining pistachio coloured solid was washed with pyridine (2 × 10 mL). Yield = 1.47 g (82 %). X-ray quality crystals were obtained via vapour diffusion of hexanes into a solution of [UO_2Cl_2(THF)$_2$] and [UI_3(dioxane)$_{1.5}$] in pyridine at room temperature. **Elemental Analysis Calcd (%)** for $C_{40}H_{40}Cl_2I_6N_8O_2U_3$: C, 21.73; H, 1.82; N, 5.07. Found: C, 21.80; H, 1.83; N, 5.13.

Reactions of the multinuclear complexes

Reaction of 1-Sm with 18-crown-6; synthesis of [{UO_2(18-crown-6)}$_2$(SmI_4)]I (5-Sm)

Method a: THF (60 mL) was added to a 100 mL Schlenk flask containing [UO_2Cl_2(THF)$_2$] (65.3 mg, 0.135 mmol), [SmI_2(THF)$_2$] (92.9 mg, 0.168 mmol) and 18-crown-6 (44.5 mg, 0.168 mmol) via cannula and the reaction mixture was stirred overnight at room temperature, resulting in the precipitation of a beige solid. The beige solid was isolated by filtration and dried under reduced pressure and characterised as [{UO_2(18-crown-6)}$_2$(SmI_4)]I (**5-Sm**). Yield = 62.7 mg (50 %). **Elemental Analysis Calcd (%)** for $C_{24}H_{48}I_5O_{16}SmU_2$: C, 15.56; H, 2.61. Found: C, 15.45; H, 2.67. **IR:** ν(asym. UO_2^+) = 834 cm^{-1} (nujol). Dissolution in pyridine releases free 18-cr-6, and the complex decomposes slowly on contact with CH_2Cl_2 in which it is only sparingly soluble, so an NMR spectrum was not obtained.

Method b: THF (60 mL) was added into a 100 mL Schlenk flask containing **1-Sm** (309 mg, 0.146 mmol) and 18-crown-6 (96.2 mg, 0.364 mmol) via cannula and the reaction mixture was stirred for 3

days at room temperature, resulting in precipitation of a beige solid. The beige solid was isolated by filtration and dried under reduced pressure. Yield = 102 mg (38 %). **Elemental Analysis Calcd (%)** for $C_{24}H_{48}I_5O_{16}SmU_2$: C, 15.56; H, 2.61. Found: C, 15.60; H, 2.67. **IR:** ν(asym. UO_2^+) = 836 cm^{-1} (nujol).

The ^1H NMR spectrum of a d_5-pyridine solution of **5-Sm** (15.4 mg, $8.31×10^{-3}$ mmol, ~0.6 mL) and $Si(SiMe_3)_4$ (5.3 mg, $1.7×10^{-2}$ mmol; internal standard) shows by integration that the solvent has displaced the 18-crown-6. ^1H NMR (d_5-pyridine, 298 K, 500 MHz): δ 3.63 (s, 24H, 18-crown-6), 0.25 (s, 36H, $Si(SiMe_3)_4$). **5-Sm** is insoluble in all other common polar aprotic solvents tried (d_8-THF, CD_2Cl_2, C_6D_6, d_8-toluene), but spectra of samples in these contain no resonances, supporting the formulation of **5-Sm**. It is known that this crown ether is readily displaced from U(VI) uranyl crown ether complexes by competing donors.[7]

Reaction of 1-Sm with KO^{t}Bu; synthesis of $[\{UO_2(py)_5\}_2(KI_2(py)_2)]_n$

Pyridine (3 mL) was added to a 7 mL vial containing **1-Sm** (148 mg, $6.97×10^{-2}$ mmol) and KO^{t}Bu (15.7 mg, 0.140 mmol), which resulted in immediate precipitation of both a white and an orange solid; the reaction was stirred for 3 hours at room temperature. The mother liquors were then decanted and the remaining orange solid was dried under reduced pressure. Yield = 62.4 mg (40 %).

Reaction of 1-Sm with $KN(SiMe_3)_2$; synthesis of $[\{UO_2(py)_5\}_2(KI_2(py)_2)]_n$

Pyridine (2 mL) was added to a 7 mL vial containing **1-Sm** (39.1 mg, $1.85×10^{-2}$ mmol) and $KN(SiMe_3)_2$ (14.8 mg, $7.42×10^{-2}$ mmol), which resulted in the immediate precipitation of both a white and an orange solid. The solution was filtered, and the filtrate was allowed to stand at room temperature overnight. The mother liquors were then decanted and the remaining orange solid was dried under reduced pressure. Yield = ~5 mg (48 %).

Crystallographic Details

General X-ray experimental details

The molecular structures of **1-Sm·3py**, **1-Dy·3py** and **4·4py** were solved using SHELXT[8] and least-square refined using SHELXL[9] in Olex2.[10] **2-Sm** and **3-Dy·4py** were solved using SuperFlip in SHELXS-97,[11] and were refined using a full-matrix least-square refinement on $|F|^2$ using SHELXL-97[11] in the WinGX suite.[12] Hydrogen atoms were treated by constrained refinement.

One lattice pyridine molecule (N(7), C(31)-C(35)) and the iodide anion in **1-Sm·3py** are occupationally disordered over overlapping positions, therefore their occupancy was set to 0.5. One lattice pyridine molecule (N(6), C(26)-C(30)) and the iodide anion in **1-Dy·3py** are occupationally disordered over overlapping positions, therefore their occupancy was set to 0.5. One of the lattice pyridine molecules in **3-Dy·4py** (N(80), C(81)-C(85); N(90), C(91)-C(95)) is positionally disordered over two positions, therefore the occupancy of all 10 atoms was set to 0.5 with thermal parameters refined anisotropically and restrained using the SIMU command. In addition, one of the coordinated pyridine ligands in **3-Dy·4py** (N(7), C(31)-C(35)) is positionally disordered over two positions; it's thermal parameters were split into two parts with their occupancy refined in a 38:62 ratio (py-A:py-B). Furthermore, the geometry of py-A was constrained using the AFIX 66 command, and the geometry of py-B was constrained to the same geometry as py-A through the use of the SAME command. Finally, the thermal parameters of N(7A) and N(7B) were restrained to have similar thermal parameters using the SIMU command, and those of C(31A)-C(35B) were restrained to have similar thermal parameters using the SIMU command; the thermal parameters of all the disordered

atoms were refined anisotropically. The iodo ligands in the UO_2I_4 unit in **4·4py** (I(3A), I(3B), I(4A), I(4B)) are positionally disordered over two positions, so were split into two parts and refined with occupancies of 0.9:0.1 for I-A:I-B. In addition, the iodo ligand bound to U(1) (I(1A), I(1B)) is positionally disordered over two positions, and was split into two parts with occupancies of 0.9:0.1 for I-A:I-B; the thermal parameters of all iodo ligand were refined anisotropically with no restraints. Furthermore, the thermal parameters of N(2) and N(3) were constrained to be the same as one another using the EADP command.

Standard X-ray details for each complex

Table S1. Crystallographic data summary for complexes **1-Sm·3py**, **1-Dy·3py** and **2-Sm**.

Complex	$[\{UO_2(py)_5\}_2(SmI_4)]I$ (**1-Sm·3py**)	$[\{UO_2(py)_5\}_2(DyI_4)]I$ (**1-Dy·3py**)	$[(UO_2I_4)\{Sm(MeCN)_6\}]_n$ (**2-Sm**)
label in cif file	1-Sm.3py	1-Dy.3py	2-Sm
Local code	exp_1664	p16198	p17008_077orth
Chemical formula	$C_{50}H_{50}I_4N_{10}O_4SmU_2·3(C_5H_5N)·I$	$C_{50}H_{50}DyI_4N_{10}O_4U_2·3(C_5H_5N)·I$	$C_{12}H_{18}I_4N_6O_2SmU$
M_r	2353.22	2365.36	1174.30
Crystal system, space group	monoclinic, $P2_1/n$	monoclinic, $P2_1/n$	Orthorhombic, *Pnam*
Temperature (K)	173(2)	173(2)	298(2) K
a, *b*, *c* (Å)	12.1522(2), 17.1078(2), 18.4527(2)	12.2179(2), 17.1019(2), 18.4444(2)	15.7944(3), 10.2473(2), 17.3932(4)
α, β, γ (°)	90, 103.847(1), 90	90, 104.201(1), 90	90, 90, 90
V (Å^3)	3724.78 (9)	3736.15(8)	2815.1(1)
Z	2	2	4
Radiation type	Mo *Ka*	Mo *Ka*	Mo *Ka*
μ (mm^{-1})	7.24	7.43	12.213
Crystal size (mm)	0.19 × 0.13 × 0.07	0.43 × 0.24 × 0.12	0.30 × 0.16 × 0.08
Diffractometer	Xcalibur, Eos	Xcalibur, Eos	Xcalibur, Eos
Absorption correction	Analytical *CrysAlis PRO*, Agilent Technologies, Version 1.171.37.34 (release 22-05-2014 CrysAlis171 .NET) (compiled May 22 2014,16:03:01) Analytical numeric absorption correction using a multifaceted crystal model based on expressions derived by R.C. Clark & J.S. Reid. (Clark, R. C. & Reid, J. S. (1995). Acta Cryst. A51, 887-897) Empirical absorption correction using spherical harmonics, implemented in SCALE3 ABSPACK scaling algorithm.	Analytical *CrysAlis PRO* 1.171.38.42b (Rigaku Oxford Diffraction, 2015) Analytical numeric absorption correction using a multifaceted crystal model based on expressions derived by R.C. Clark & J.S. Reid. (Clark, R. C. & Reid, J. S. (1995). Acta Cryst. A51, 887-897) Empirical absorption correction using spherical harmonics, implemented in SCALE3 ABSPACK scaling algorithm.	Analytical *CrysAlis PRO* 1.171.38.42b (Rigaku Oxford Diffraction, 2015) Analytical numeric absorption correction using a multifaceted crystal model based on expressions derived by R.C. Clark & J.S. Reid. (Clark, R. C. & Reid, J. S. (1995). Acta Cryst. A51, 887-897) Empirical absorption correction using spherical harmonics, implemented in SCALE3 ABSPACK scaling algorithm.
T_{min}, T_{max}	0.3400, 0.6312	0.665, 0.863	0.1207, 0.4416
θ_{min}, θ_{max}	3.287, 28.590	3.282, 29.019	3.317, 29.924
No. of measured, independent and observed [$I > 2\sigma(I)$] reflections	84459, 8528, 7549	97459, 9596, 8057	65045, 3339, 3022
R_{int}	0.049	0.042	0.0608
$R[F^2 > 2\sigma(F^2)]$, $wR(F^2)$, *S*	0.051, 0.145, 1.09	0.045, 0.127, 1.07	0.0259, 0.0538, 1.117
No. of reflections	8528	9596	3339
No. of parameters	427	427	137
No. of restraints	0	145	0
$\Delta\rho_{max}$, $\Delta\rho_{min}$ (e Å^{-3})	1.640, –5.972	2.623, –3.956	1.379, –0.828

Table S2. Crystallographic data summary for complexes **3-Dy·4py** and **4·4py**.

Complex	$[(UO_2I_4)\{DyI(py)_5\}_2]$ (**3-Dy·4py**)	$[(UO_2I_4)\{UICl(py)_4\}_2]$ (**4·4py**)
label in cif file	3-Dy.4py	4.4py
Local code	p17020a_tri	po16003

Chemical formula	$C_{50}H_{50}Dy_2I_6O_2U\cdot3(C_5H_5N)\cdot0.5(C_{10}H_{10}N_2)$	$C_{40}H_{40}Cl_2I_6N_8O_2U_3\cdot4(C_5H_5N)$
M_r	2463.83	2527.59
Crystal system, space group	Triclinic, *P*–1	monoclinic, $P2_1/n$
Temperature (K)	173(2) K	120
a, *b*, *c* (Å)	13.0413(2), 15.3427(3), 20.2947(4)	12.5860(3), 15.6705(4), 18.8464(5)
α, β, γ (°)	86.671(2), 81.741(2), 82.841(2)	90, 105.211(2), 90
V (Å^3)	3983.96(13)	3586.8
Z	2	2
Radiation type	Mo *K*a	Mo *K*a
μ (mm^{-1})	6.258	9.460
Crystal size (mm)	0.61 × 0.36 × 0.08	0.16 × 0.15 × 0.08
Diffractometer	Xcalibur, Eos	SuperNova, Dual, Cu at zero, Atlas
Absorption correction	Analytical *CrysAlis PRO* 1.171.38.42b (Rigaku Oxford Diffraction, 2015) Analytical numeric absorption correction using a multifaceted crystal model based on expressions derived by R.C. Clark & J.S. Reid. (Clark, R. C. & Reid, J. S. (1995). Acta Cryst. A51, 887-897) Empirical absorption correction using spherical harmonics, implemented in SCALE3 ABSPACK scaling algorithm.	Gaussian *CrysAlis PRO* 1.171.38.42b (Rigaku Oxford Diffraction, 2015) Numerical absorption correction based on gaussian integration over a multifaceted crystal model Empirical absorption correction using spherical harmonics, implemented in SCALE3 ABSPACK scaling algorithm.
T_{min}, T_{max}	0.1148, 0.6344	0.565, 0.711
θ_{min}, θ_{max}	2.8767, 30.2637	3.403, 29.171
No. of measured, independent and observed [$I > 2\sigma(I)$] reflections	78601, 18230, 14658	63656, 9339, 7079
R_{int}	0.0405	0.066
$R[F^2 > 2\sigma(F^2)]$, $wR(F^2)$, S	0.0341, 0.0639, 1.045	0.056, 0.127, 1.033
No. of reflections	18230	9339
No. of parameters	929	406
No. of restraints	294	0
$\Delta\rho_{max}$, $\Delta\rho_{min}$ (e Å^{-3})	1.334, –1.058	2.798, –2.685

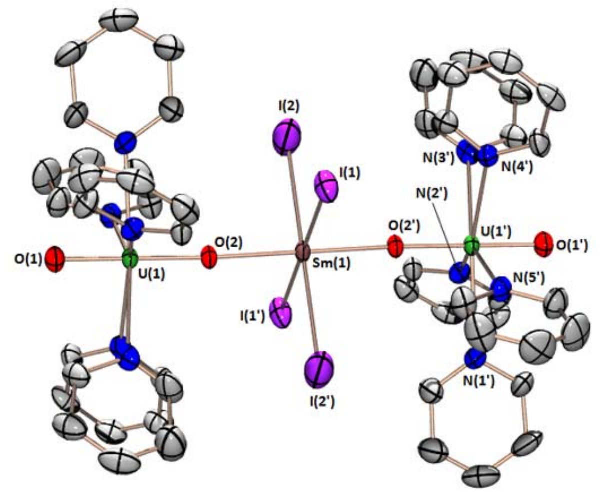

Figure S1. Solid-state structure of **1-Sm·3py** with ellipsoids drawn at 50% probability. Hydrogen atoms, lattice solvent and the iodide counter-anion are omitted for clarity. Key bond lengths [Å] and angles [°]: U(1)–O(1) = 1.801(6); U(1)–O(2) = 1.916(6); U(1)–N(1) = 2.576(7); U(1)–N(2) = 2.597(8); U(1)–N(3) = 2.588(7); U(1)–N(4) = 2.626(8); U(1)–N(5) = 2.584(7); Sm(1)–I(1) = 2.993(1); Sm(1)–I(2) = 3.1135(6); Sm(1)–O(2) = 2.330(6); O(1)–U(1)–O(2) = 177.7(3). O(2)–Sm(1)–O(2') = 180.0(3); N(1)–U(1)–N(2) = 71.0(3); N(2)–U(1)–N(3) = 71.8(2); N(3)–U(1)–N(4) = 70.5(2); N(4)–U(1)–N(5) = 72.2(2); I(1)–Sm(1)–I(2) = 89.39(3); I(2)–Sm(1)–I(1') = 90.61(3); I(1')–Sm(1)–I(2') = 89.39(3); I(2')–Sm(1)–I(1) = 90.61(3); I(1)–Sm(1)–I(1') = 180.0; I(2)–Sm(1)–I(2') = 180.0.

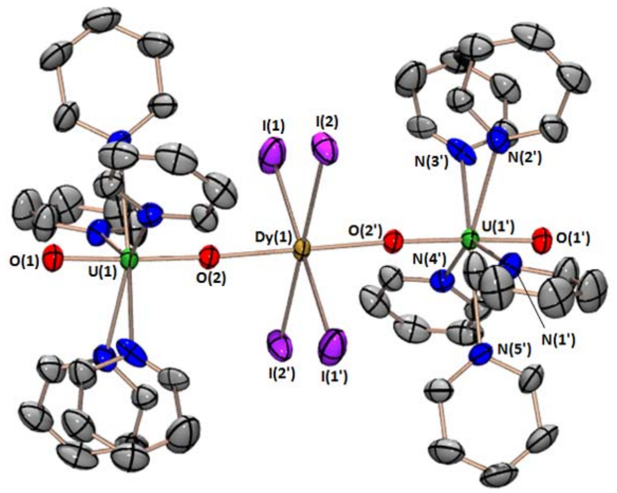

Figure S2. Solid-state structure of **1-Dy·3py** with ellipsoids drawn at 50% probability. Hydrogen atoms, lattice solvent and the iodide counter-anion are omitted for clarity. Key bond lengths [Å] and angles [°]: U(1)–O(1) = 1.808(5); U(1)–O(2) = 1.919(5); U(1)–N(1) = 2.586(6); U(1)–N(2) = 2.582(6); U(1)–N(3) = 2.625(7); U(1)–N(4) = 2.584(6); U(1)–N(5) = 2.584(6); Dy(1)–I(1) = 2.9992(7); Dy(1)–I(2) = 3.0655(5); Dy(1)–O(2) = 2.270(5); O(1)–U(1)–O(2) = 177.6(2); O(2)–Dy(1)–O(2') = 180.0; N(1)–U(1)–N(2) = 71.8(2); N(2)–U(1)–N(3) = 70.43(19); N(3)–U(1)–N(4) = 71.77(19); N(4)–U(1)–N(5) = 73.82(19); I(1)–Dy(1)–I(2) = 90.46(2); I(2)–Dy(1)–I(1') = 89.54; I(1')–Dy(1)–I(2') = 90.46(2); I(2')–Dy(1)–I(1) = 89.54; I(1)–Dy(1)–I(1') = 180.0; I(2)–Dy(1)–I(2') = 180.0.

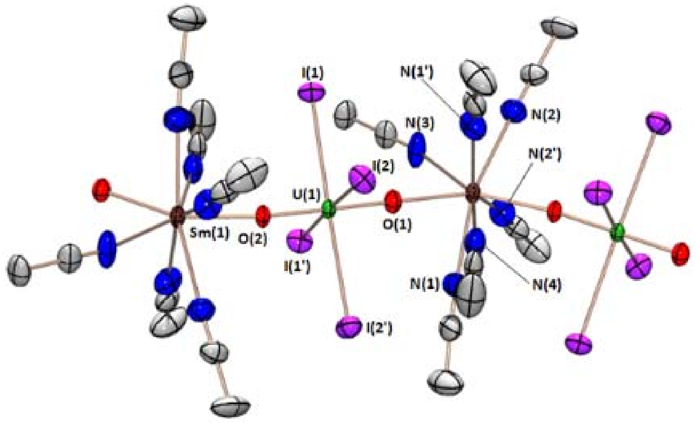

Figure S3. Solid-state structure of **2-Sm** with ellipsoids drawn at 50% probability. Hydrogen atoms and lattice solvent are omitted for clarity. Key bond lengths [Å] and angles [°]: U(1)–O(1) = 1.868(5); U(1)–O(2) = 1.883(4); Sm(1)–O(1) = 2.351(5); Sm(1)–O(2) = 2.318(4); U(1)–I(1) = 3.1007(4); U(1)–I(2) = 3.1313(4); Sm(1)–N(1) = 2.518(6); Sm(1)–N(2) = 2.563(5); Sm(1)–N(3) = 2.556(7); Sm(1)–N(4) = 2.507(7). O(1)–U(1)–O(2) = 179.3(2); O(1)–Sm(1)–O(2) = 145.62(17); I(1)–U(1)–I(2) = 89.412(11); I(2)–U(1)–I(2') = 88.379(17); I(2')–U(1)–I(1) = 89.412(11); I(1)–U(1)–I(1') = 92.786(16); I(1)–U(1)–I(2') = 177.668(12).

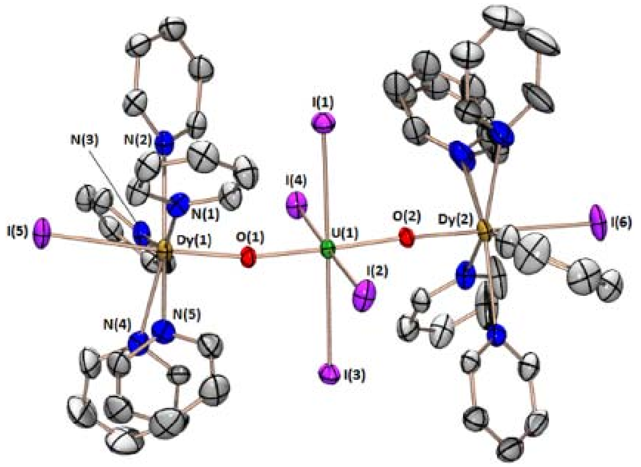

Figure S4. Solid-state structure of **3-Dy·4py** with ellipsoids drawn at 50% probability. Hydrogen atoms and lattice solvent omitted for clarity. One of the pyridine ligands coordinated to Dy(2) is disordered over two positions (N(6), C(31), C(32), C(33), C(34), C(35); occupancy of orientation A:B is equal to 0.38:0.62); both orientations are shown. Key bond lengths [Å] and angles [°]:U(1)–O(1) = 2.058(3); U(1)–O(2) = 2.068(3); U(1)–I(1) = 3.1504(4); U(1)–I(2) = 3.1588(4); U(1)–I(3) = 3.1618(4); U(1)–I(4) = 3.1425(4); Dy(1)–O(1) = 2.126(3); Dy(1)–I(5) = 3.0914(4); Dy(1)–N(1) = 2.557(4); Dy(1)–N(2) = 2.545(5); Dy(1)–N(3) = 2.575(4); Dy(1)–N(4) = 2.537(5); Dy(1)–N(5) = 2.542(4); Dy(2)–O(2) = 2.119(3), Dy(2)–I(6) = 3.1109(4); Dy(2)–N(6) = 2.566(5); Dy(2)–N(7) = 2.547(15)/2.514(12); Dy(2)–N(8) = 2.544(4); Dy(2)–N(9) = 2.519(5); Dy(2)–N(10) = 2.591(4). O(1)–U(1)–O(2) = 177.67(14); I(5)–Dy(1)–O(1) = 176.59(9); I(6)–Dy(2)–O(2) = 178.31(9); Dy(1)–O(1)–U(1) = 170.47(19); Dy(2)–O(2)–U(1) = 173.61(18); I(1)–U(1)–I(2) = 91.43(9); I(2)–U(1)–I(3) = 88.135(11); I(3)–U(1)–I(4) = 91.565(11); I(4)–U(1)–I(1) = 90.235(11); I(1)–U(1)–I(3) = 177.569(11); I(2)–U(1)–I(4) = 178.421(11); N(1)–Dy(1)–N(2) = 75.01(14); N(2)–Dy(1)–N(3) = 72.56(15); N(3)–Dy(1)–N(4) = 70.10(15); N(4)–Dy(1)–N(5) = 70.02(14); N(5)–Dy(1)–N(1) = 71.30(15); N(6)–Dy(2)–N(7) = 71.8(10)/ 68.0(7); N(7)–Dy(2)–N(8) = 69.7(10)/74.0(7); N(8)–Dy(2)–N(9) = 72.59(15); N(9)–Dy(2)–N(10) = 70.77(14); N(10)–Dy(2)–N(6) = 74.24(16).

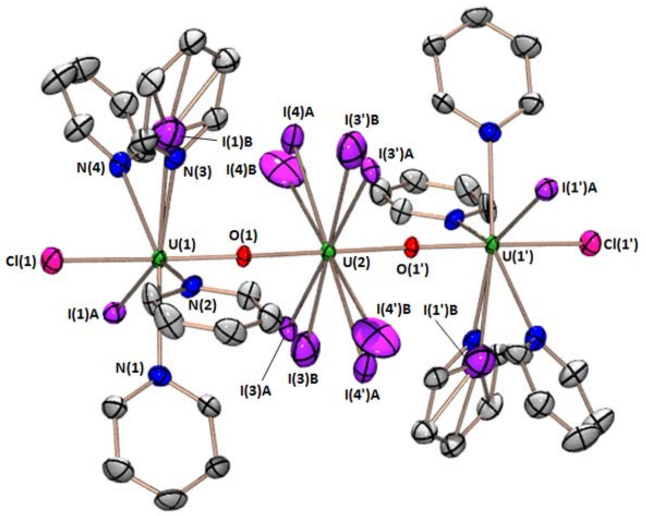

Figure S5. Solid-state structure of **4·4py** with ellipsoids drawn at 50% probability. Hydrogen atoms and lattice solvent are omitted for clarity. The iodo ligands bound to U(2), and I(1) and the N(3)-pyridine ligand are disordered over two positions; key bond lengths [Å] and angles [°] are given for the orientation of highest occupancy (A = 0.9, B = 0.1). Note: Refinement of the B-orientation of the N(3)-pyridine ligand was unstable, therefore it was not modelled in the final solution. U(1)–O(1) = 2.042(5); U(2)–O(1) = 2.166(5); U(1)–N(1) = 2.578(7); U(1)–N(2) = 2.609(7); U(1)–N(3) = 2.56(1); U(1)–N(4) = 2.632(8); U(1)–I(1) = 3.0679(8); U(1)–Cl(1) = 2.640(2); U(2)–I(3) = 3.0572(8); U(2)–I(4) = 3.0436(8); Cl(1)–U(1)–O(1) = 173.9(2); O(1)–U(2)–O(1') = 180.0; N(1)–U(1)–N(2) = 67.0(2); N(2)–U(1)–N(3) = 70.4(3); N(3)–U(1)–N(4) = 69.1(3); N(4)–U(1)–I(1) = 76.2(2); I(3)–U(2)–I(4) = 88.51(3); I(4)–U(2)–I(3') = 91.49(3); I(3')–U(2)–I(4') = 88.51(3); I(4')–U(2)–I(3) = 91.49(3); I(3)–U(2)–I(3') = 180.0; I(4)–U(2)–I(4') = 180.0.

The susceptibility for **1-Sm** is essentially featureless, the χT *vs* T plot for **4** shows a clear upturn between 8 and 6 K followed by a sharp drop upon further decreasing the temperature. Such an upturn is usually considered as a signature of ferromagnetic coupling, which is extremely unusual in oxygen-bridged U(IV) complexes. However, an alternative possibility is that each of the two outer U(IV) interacts antiferromagnetically with the central one, whilst also carrying a larger magnetic moment. We favour the latter hypothesis in this case, because fitting the high-temperature part of the inverse susceptibility of **4** (

6) with a Curie-Weiss law $\chi^{-1} = 8(T - \Theta)/\mu_{eff}^2$ gives $\mu_{eff}^2 = 21.4\ \mu_B^2$ and $\Theta = -36.2$ K; the negative Curie-Weiss temperature Θ usually indicates the presence of antiferromagnetic interactions, whereas the obtained value for the effective magnetic moment is only slightly larger than that corresponding to two U(IV) ions and definitely lower than for three.

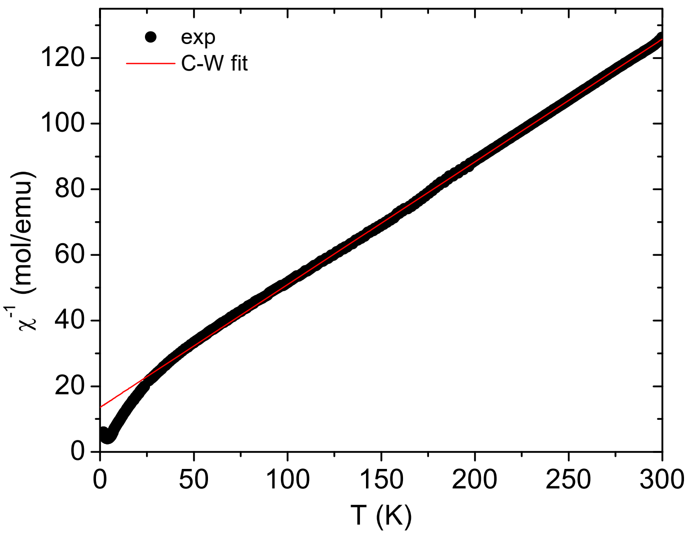

Figure S6: inverse dc magnetic susceptibility χ^{-1} as a function of temperature T plotted for **4**. The filled black dots are the experimental points, the red line is the fit to a Curie-Weiss law (see the main text for more details).

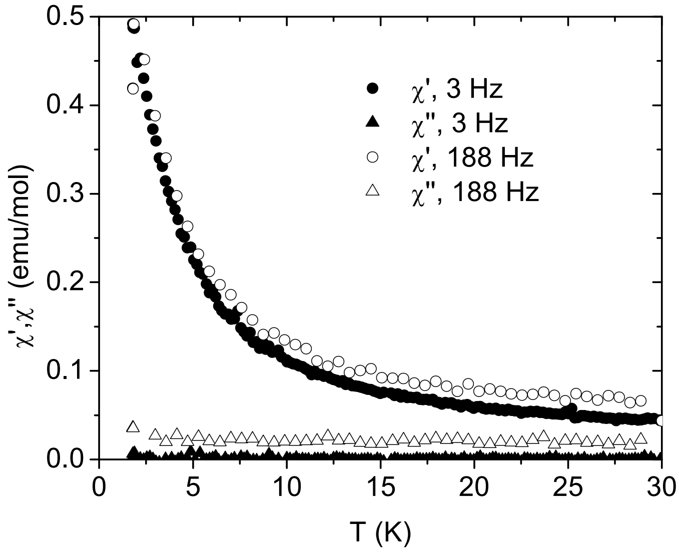

Figure S7: Zero-field ac magnetic susceptibility of **4** plotted as a function of temperature T.

The ac magnetic susceptibility of **4** was measured at various temperatures, frequencies and fields. Unfortunately we did not find any significant slowing down of the magnetic relaxation processes. The zero-field data are presented in Figure S7; ac magnetic susceptibility curves measured for **4** without external dc magnetic field.

FTIR and Raman Spectra

General Details: IR samples were mulled in nujol. Raman spectra were recorded on powders sealed in melting point capillaries using either a 785nm laser (100 % laser power) or 514nm (10 % power, 100 % focus or 50 % power, 25 % focus; 100 % power showed sample degradation after 1 scan).

Some of the data are presented without assignment as it is not clear whether sample degradation is occurring in the Raman experiments. The symmetric stretch is anticipated to be located at lower energy than the associated antisymmetric stretch, but in some cases spectra of analytically pure material are either blank or complicated. Where assignments have been made, these are tentative, and have been made by comparison with literature precedents.

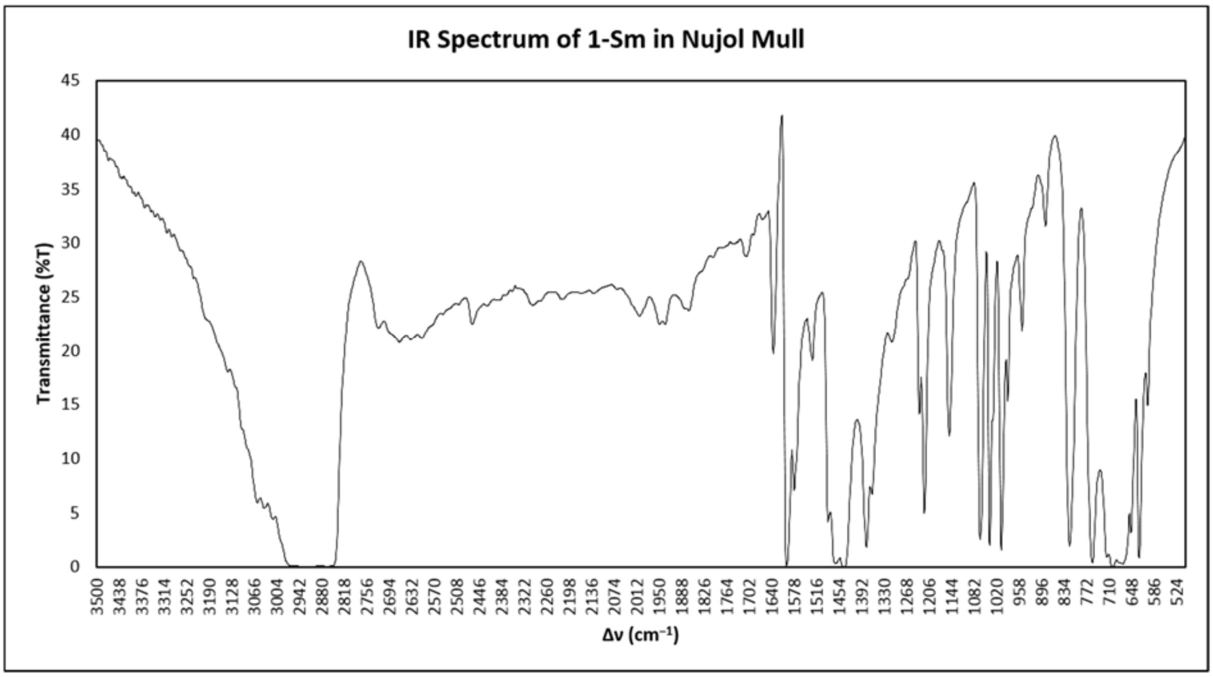

Figure S8. IR spectrum of **1-Sm** (method a) in nujol mull.

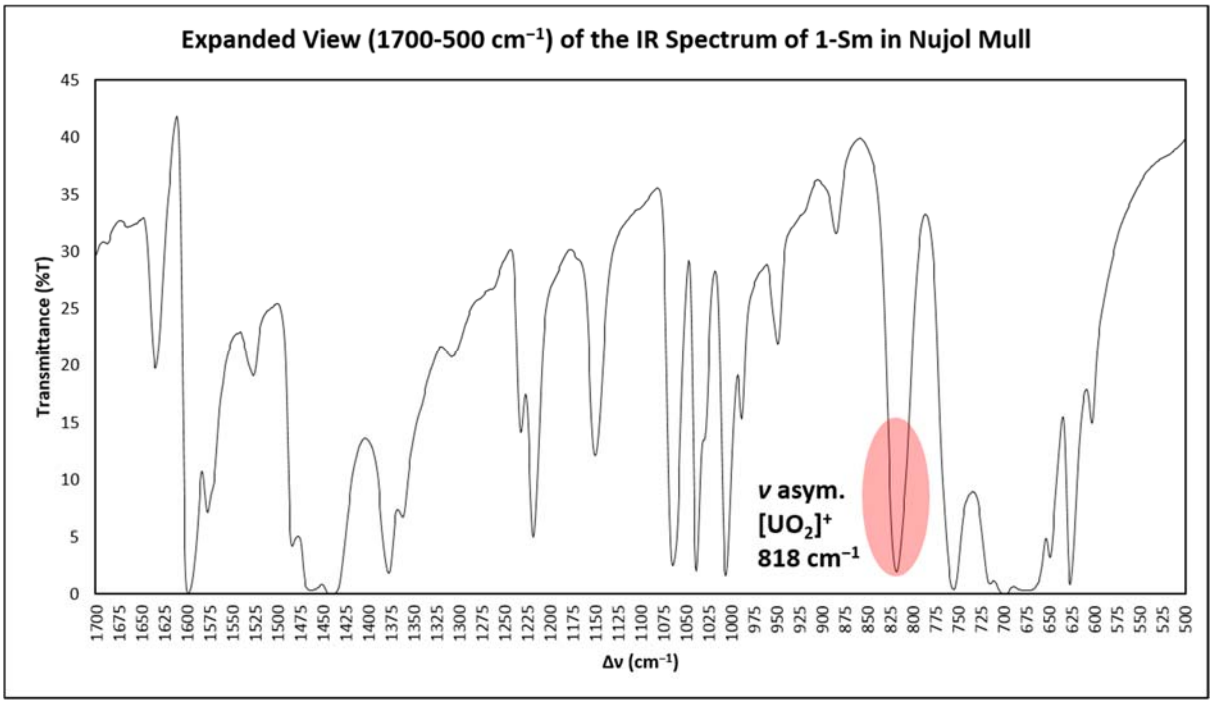

Figure S9. Expansion of the IR spectrum of **1-Sm** in nujol mull. The antisymmetric stretch, $v_3(UO_2)$ is tentatively assigned as 818 cm^{-1}.

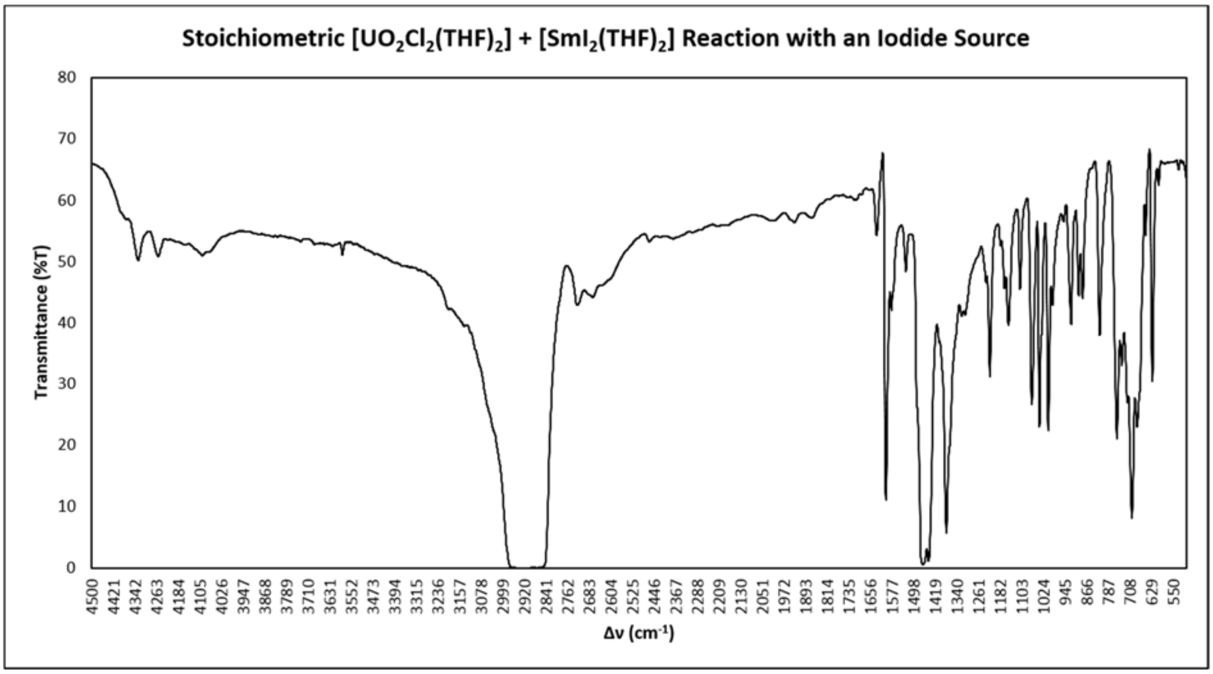

Figure S10. IR spectrum of **1-Sm** made by method b from stoichiometric U/Sm and an external iodide source. nujol mull.

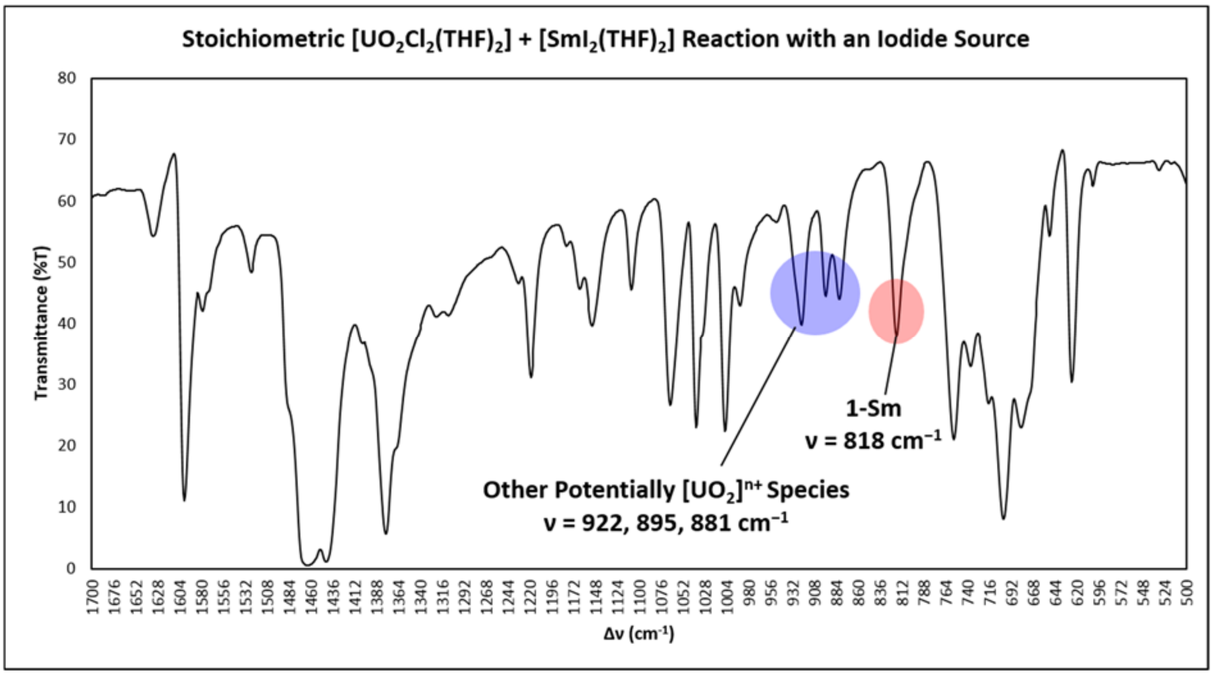

Figure S11. Expansion of the IR spectrum of **1-Sm** made by method b from stoichiometric U/Sm and an external iodide source. nujol mull.

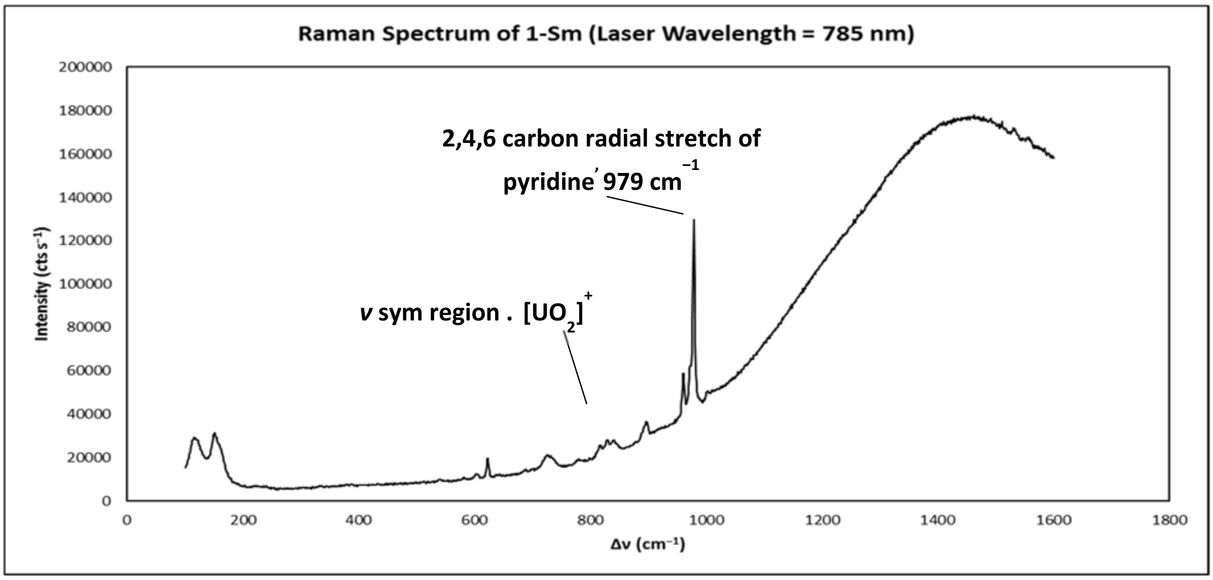

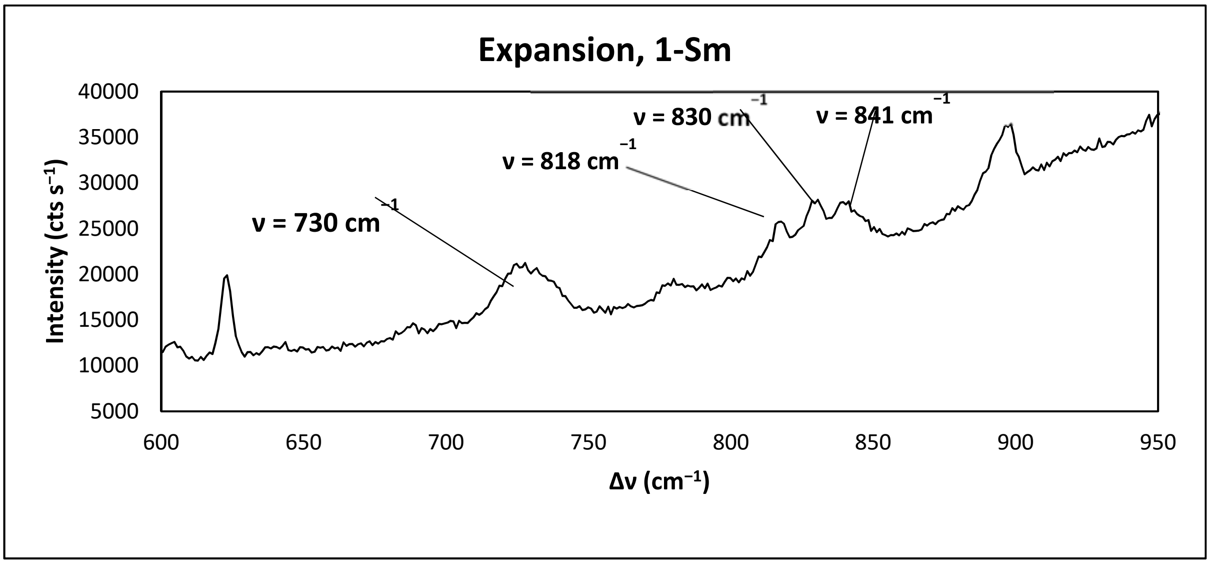

Figure S12. Raman spectrum of **1-Sm**. Scanned from 1600-100 cm^{-1} (upper) and 950-600 cm^{-1} (lower). laser wavelength = 785 nm, laser focus = 100%, laser power = 100%, exposure = 25 seconds, scans = 15.

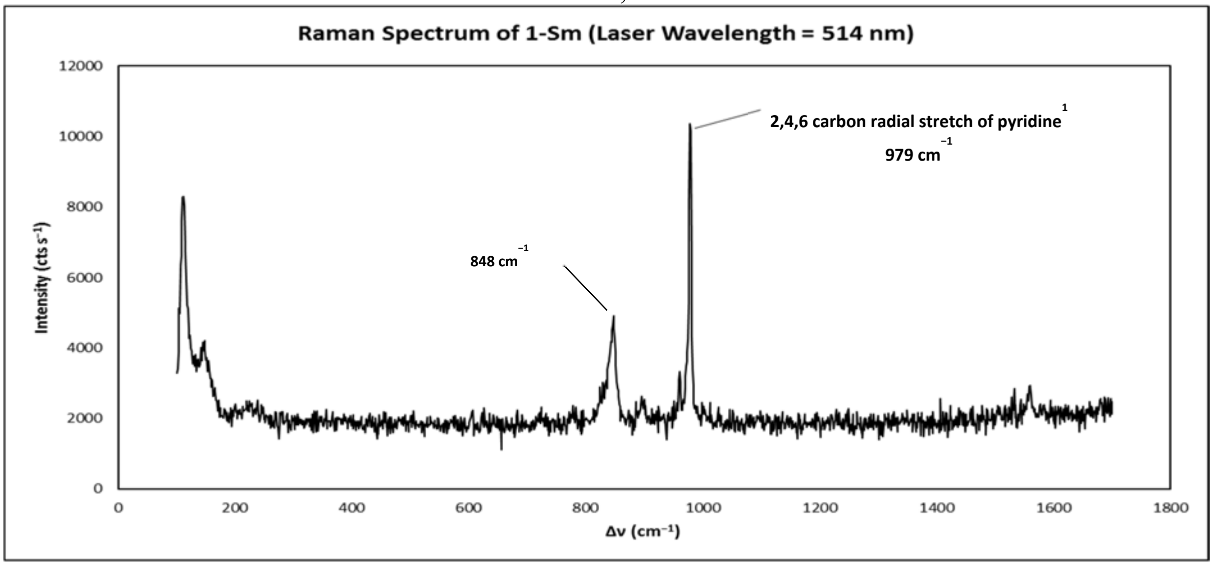

Figure S13. Raman spectrum of **1-Sm**. Scanned from 1700-100 cm^{-1}, laser wavelength = 514 nm, laser focus = 100%, laser power = 10%, exposure = 25 seconds, scans = 15. It is unclear whether the sample has decomposed.

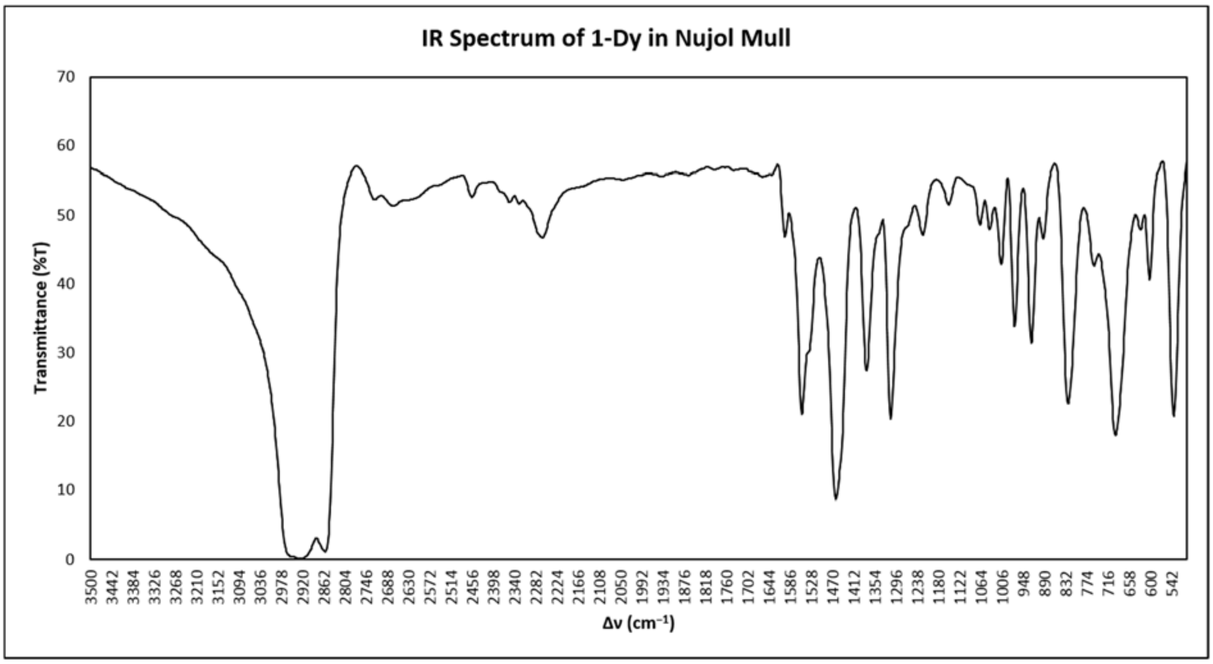

Figure S14. IR spectrum of **1-Dy** in nujol mull.

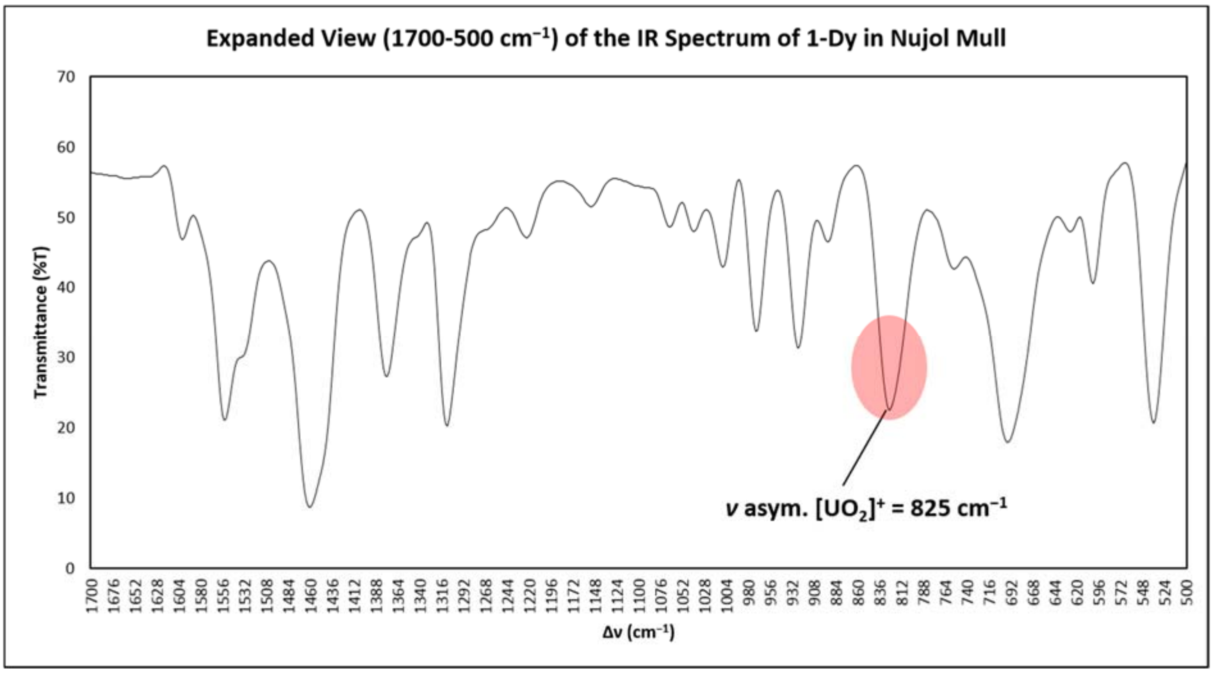

Figure S15. Expansion of the IR spectrum of **1-Dy** in nujol mull. The antisymmetric stretch, $v_3(UO_2)$ is tentatively assigned as 825 cm^{-1})

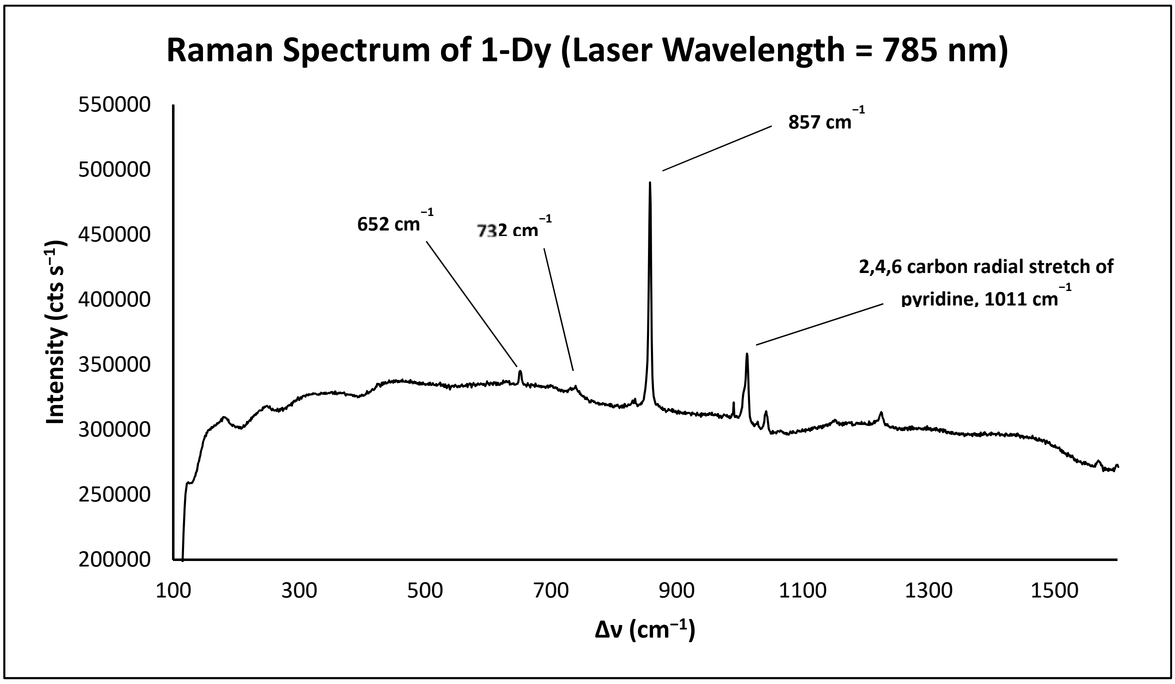

Figure S16. Raman spectrum of **1-Dy**. Scanned from 1600-100 cm^{-1}, laser wavelength = 785 nm, laser focus = 100%, laser power = 100%, exposure = 25 seconds, scans = 15.

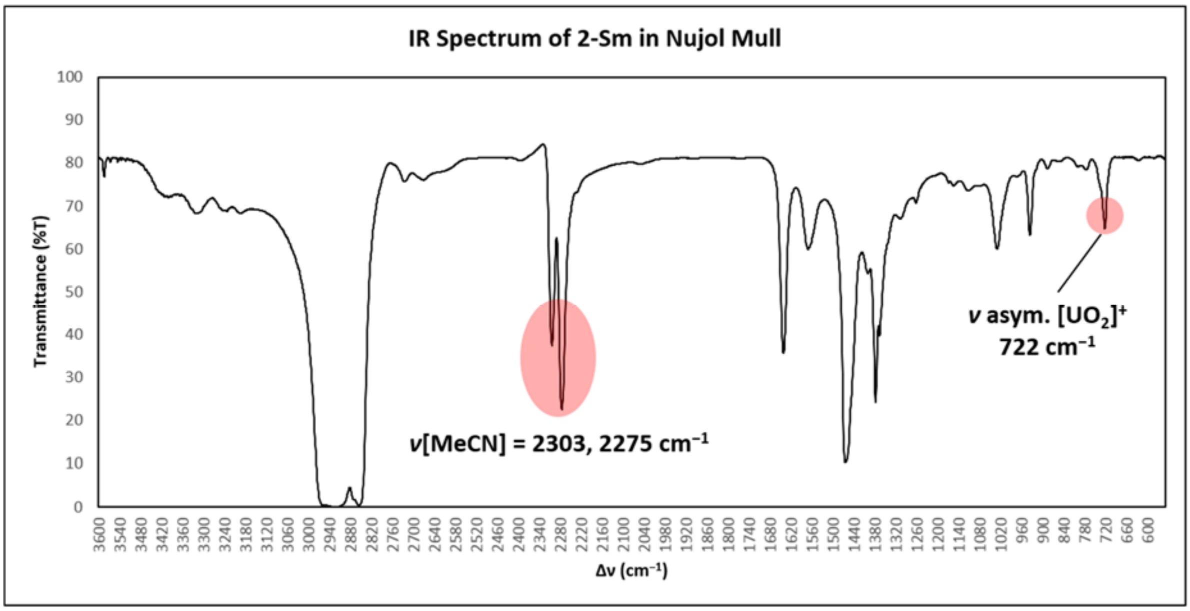

Figure S17. IR spectrum of **2-Sm** in nujol mull.

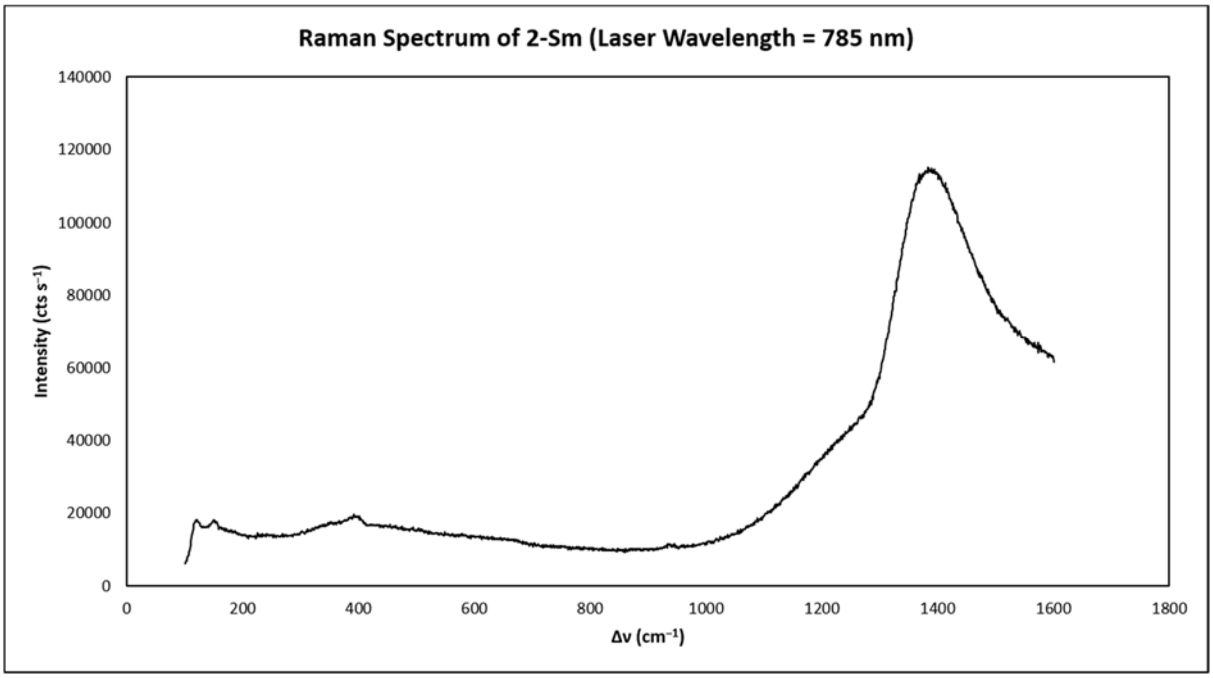

Figure S18. Raman spectrum of **2-Sm**. Scanned from 1600-100 cm^{-1}, laser wavelength = 785 nm, laser focus = 100%, laser power = 50%, exposure = 45 seconds, scans = 15.

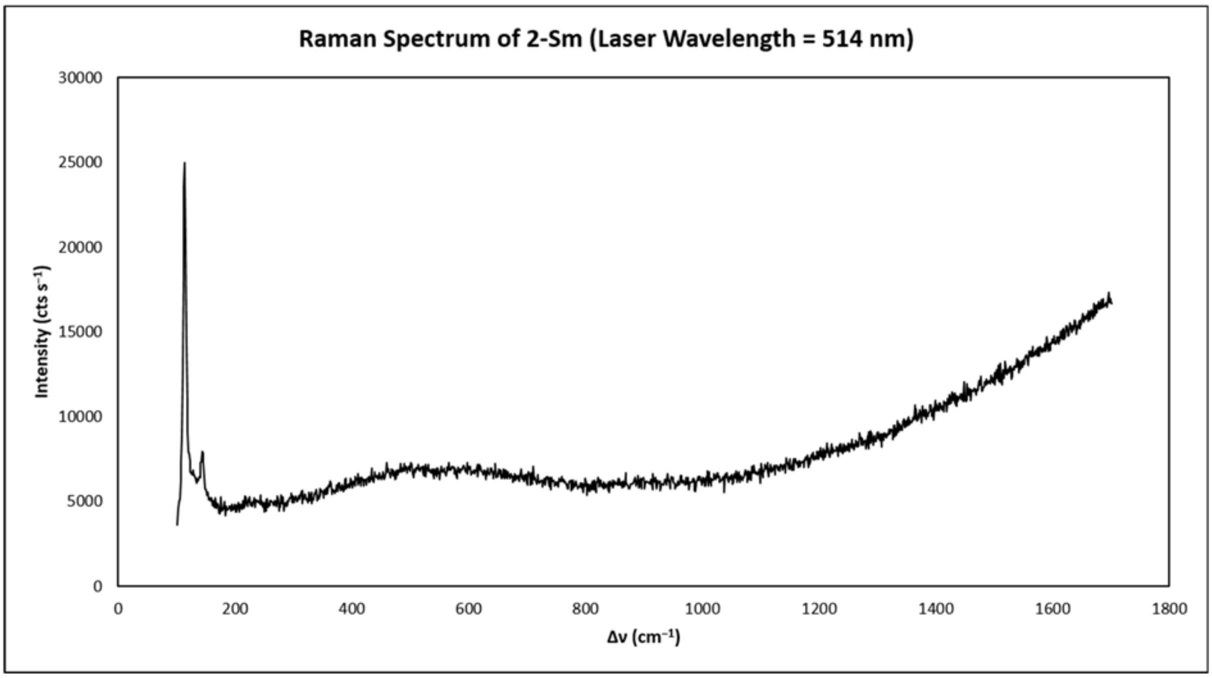

Figure S19. Raman spectrum of **2-Sm**. Scanned from 1700-100 cm^{-1}, laser wavelength = 514 nm, laser focus = 25%, laser power = 50%, exposure = 25 seconds, scans = 15.

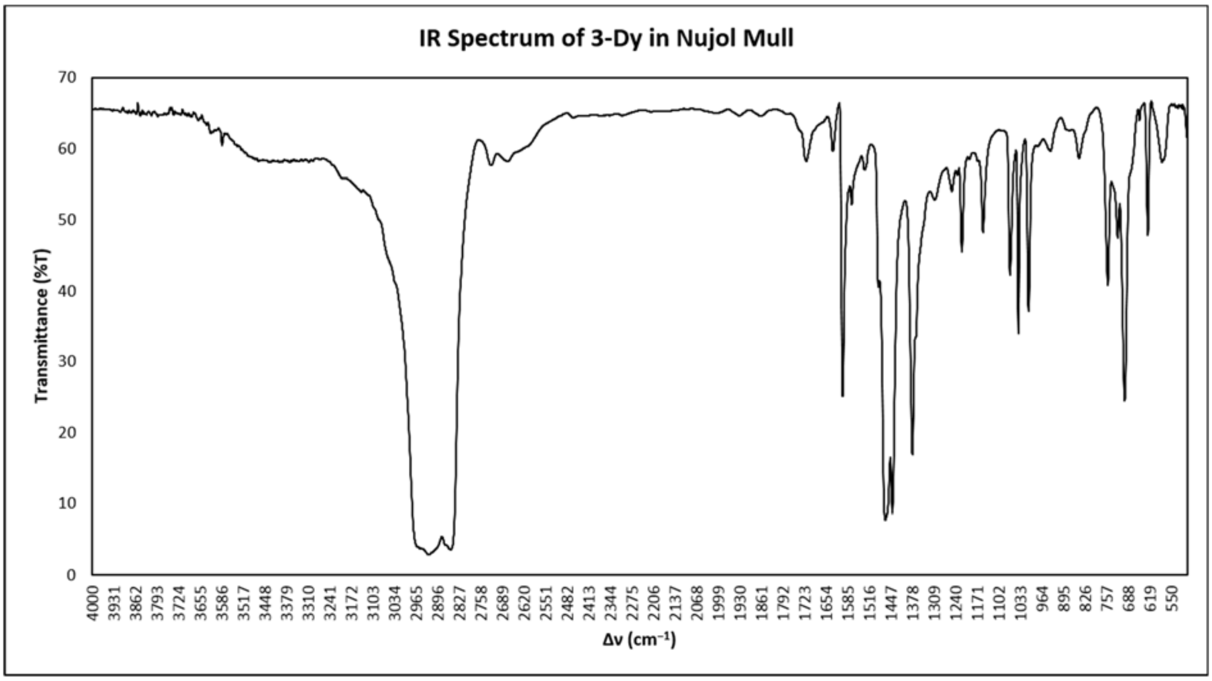

Figure S20. IR spectrum of **3-Dy** in nujol mull.

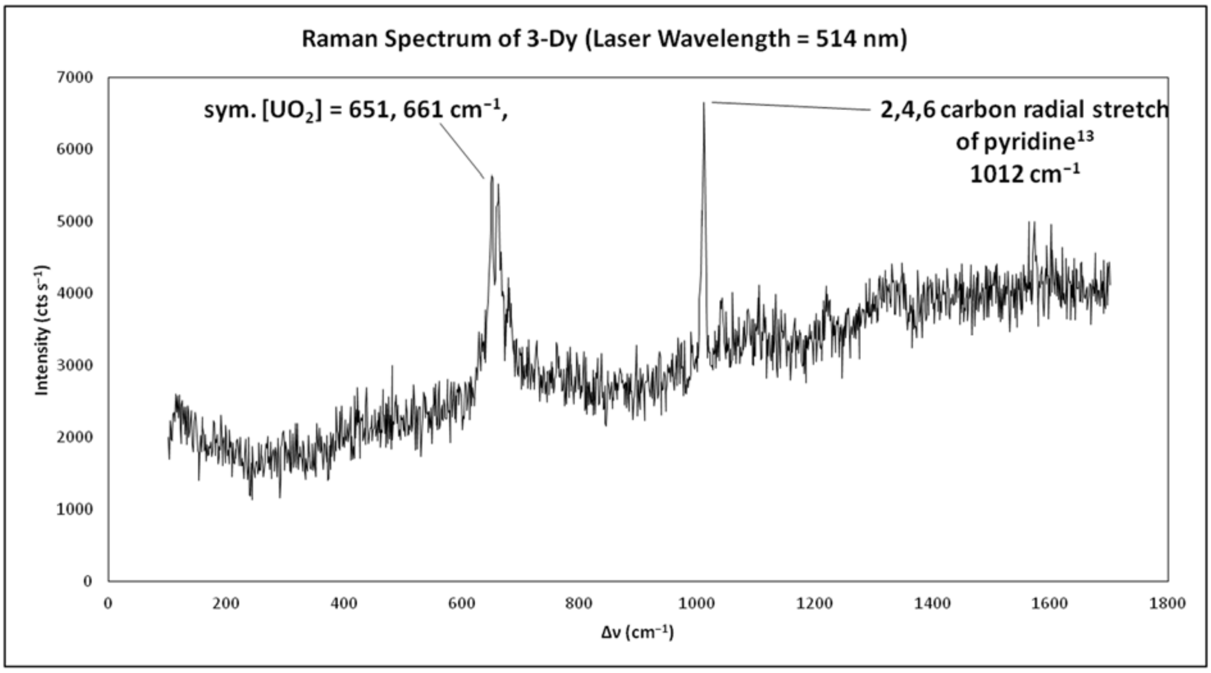

Figure S21. Raman spectrum of **3-Dy**. Scanned from 1700-100 cm^{-1}, laser wavelength = 514 nm, laser focus = 25%, laser power = 50%, exposure = 25 seconds, scans = 15.

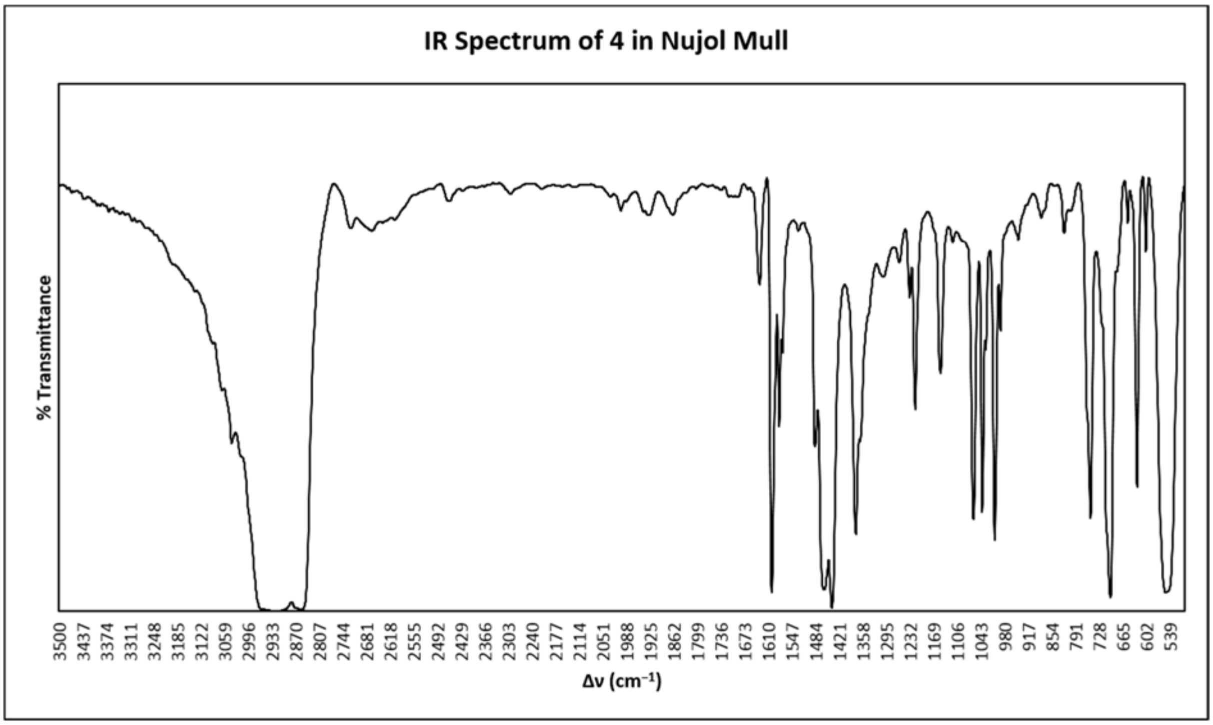

Figure S22. IR spectrum of **4** in nujol mull.

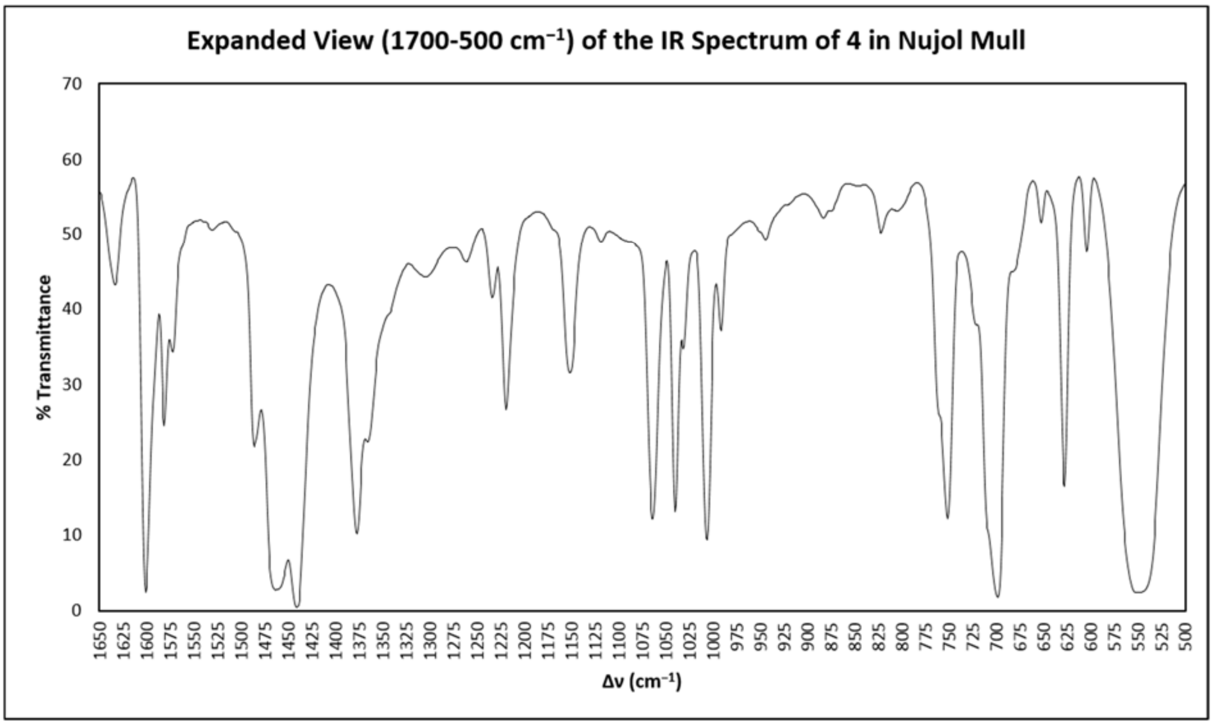

Figure S23. Expansion of the IR spectrum of **4** in nujol mull.

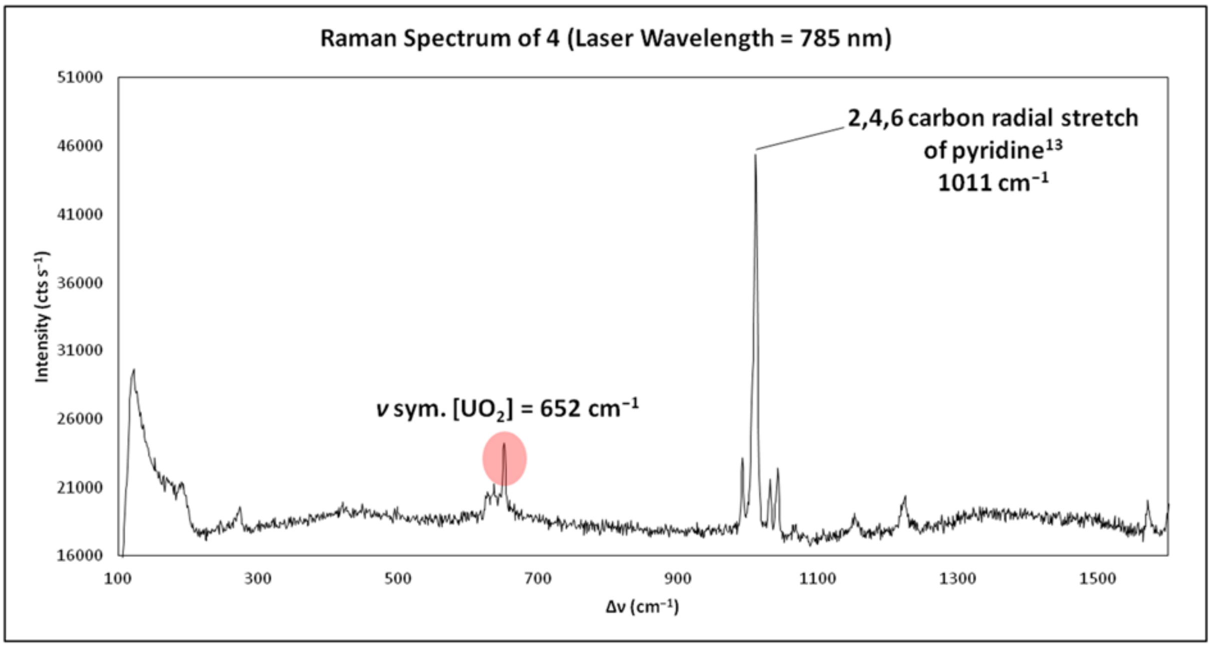

Figure S24. Raman spectrum of **4**. Scanned from 1600-100 cm^{-1}, laser wavelength = 785 nm, laser focus = 100%, laser power = 50%, exposure = 45 seconds, scans = 15. Tentative assignment of v_{symm} is labelled.

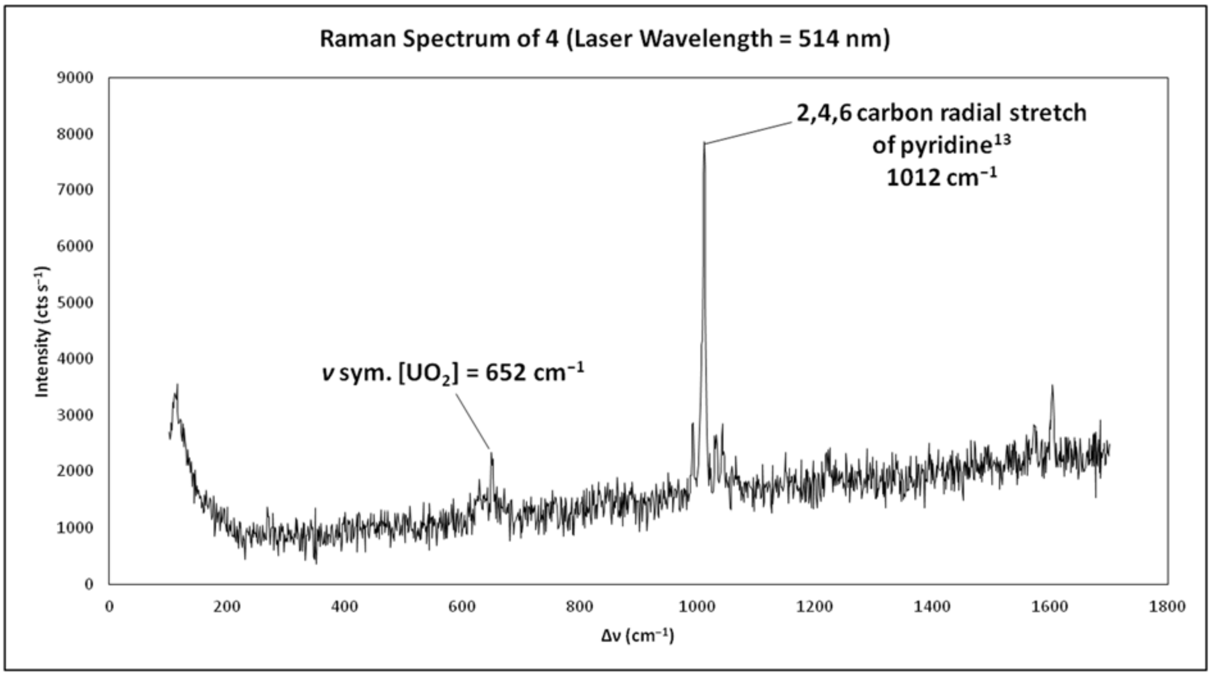

Figure S25. Raman spectrum of **4**. Scanned from 1700-100 cm^{-1}, laser wavelength = 514 nm, laser focus = 25%, laser power = 50%, exposure = 25 seconds, scans = 15.

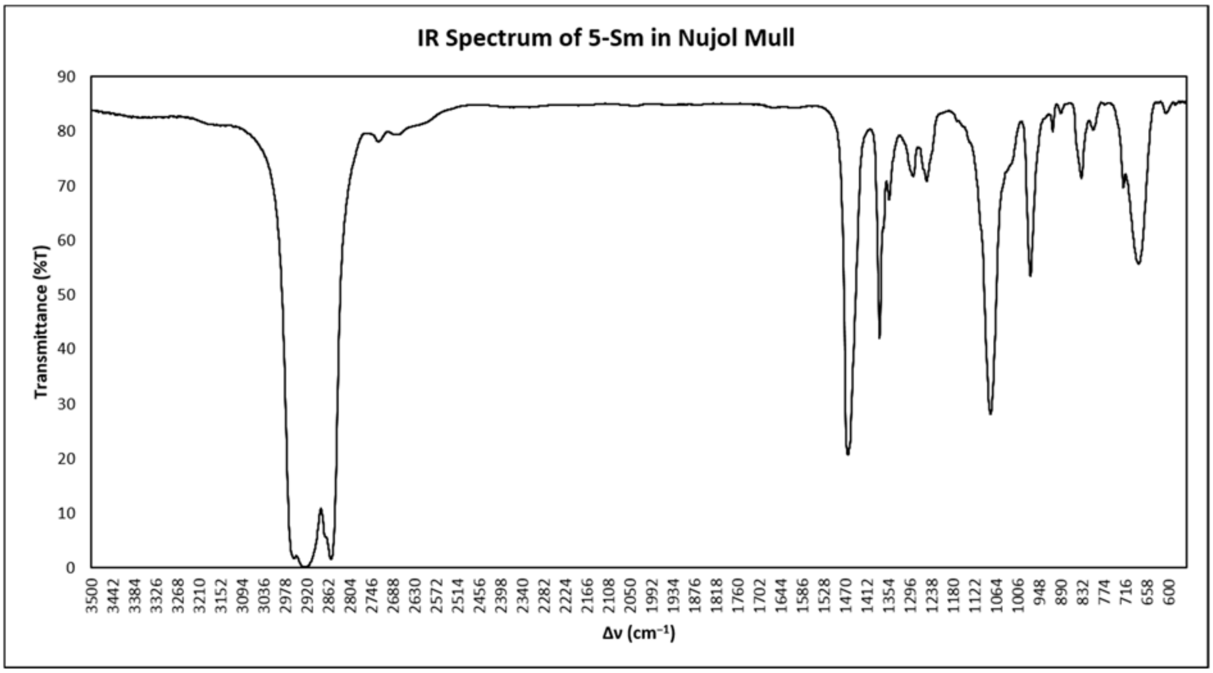

Figure S26. IR spectrum of **5-Sm** prepared via Method *a* in nujol mull. Tentative assignment of ν_{asymm} is labelled.

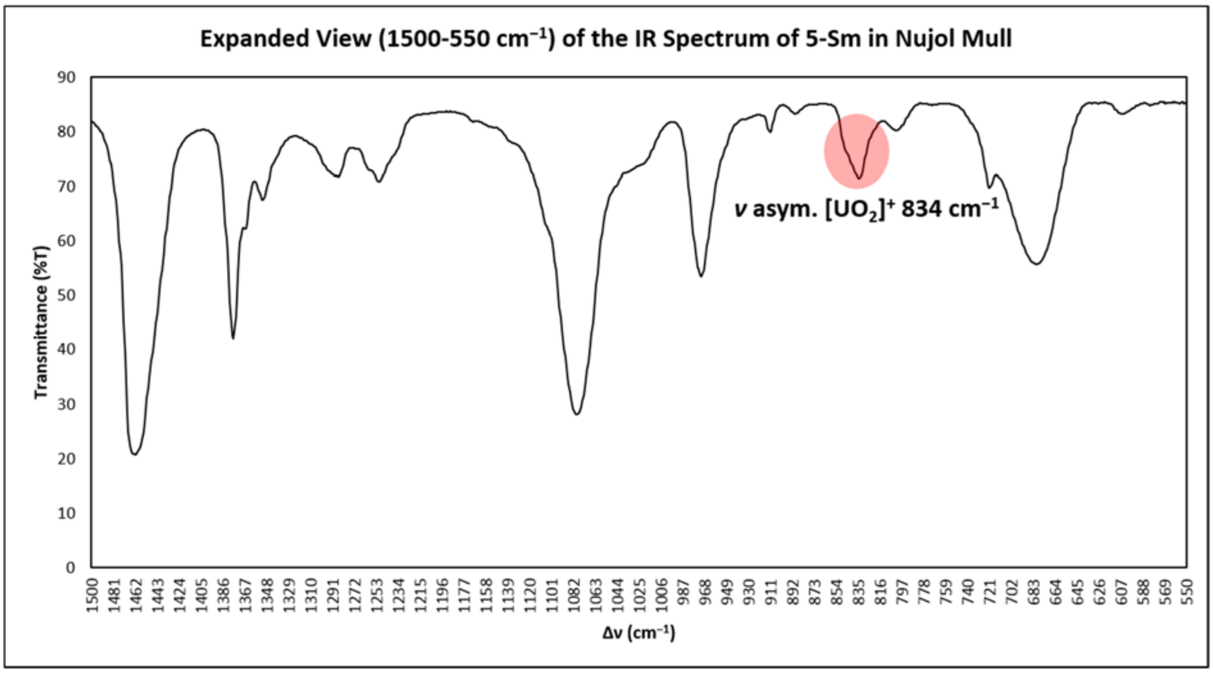

Figure S27. Expansion of the IR spectrum of **5-Sm** prepared via Method *a* in nujol mull.

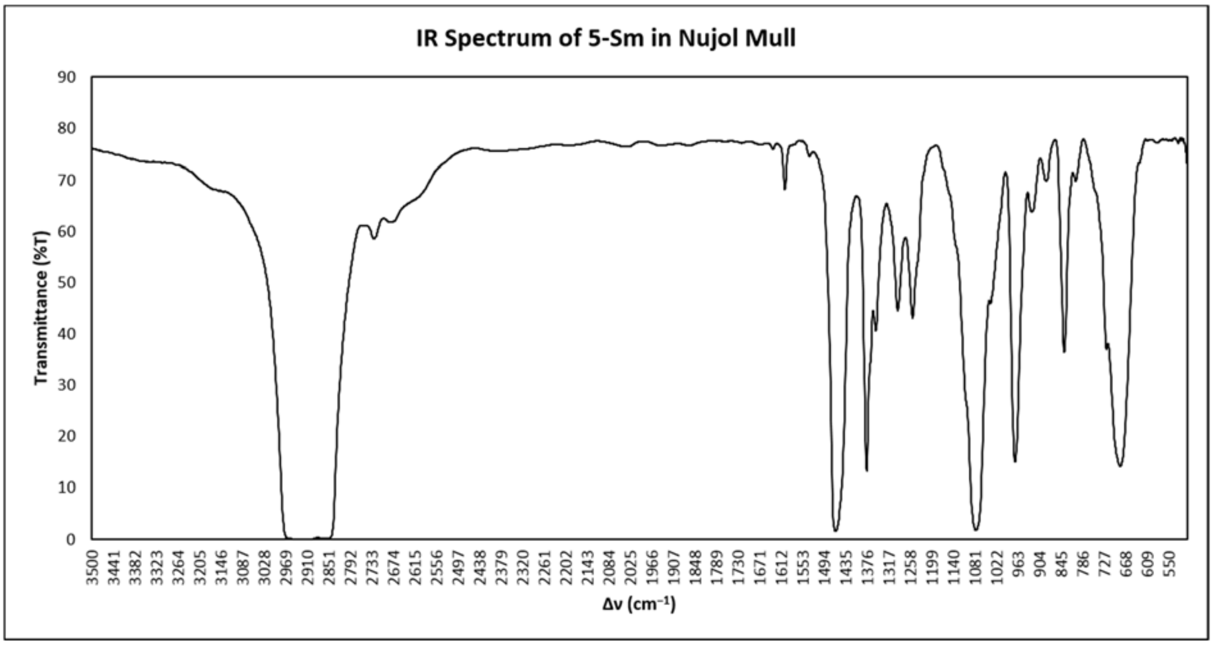

Figure S28. IR spectrum of **5-Sm** prepared via Method *b* in nujol mull.

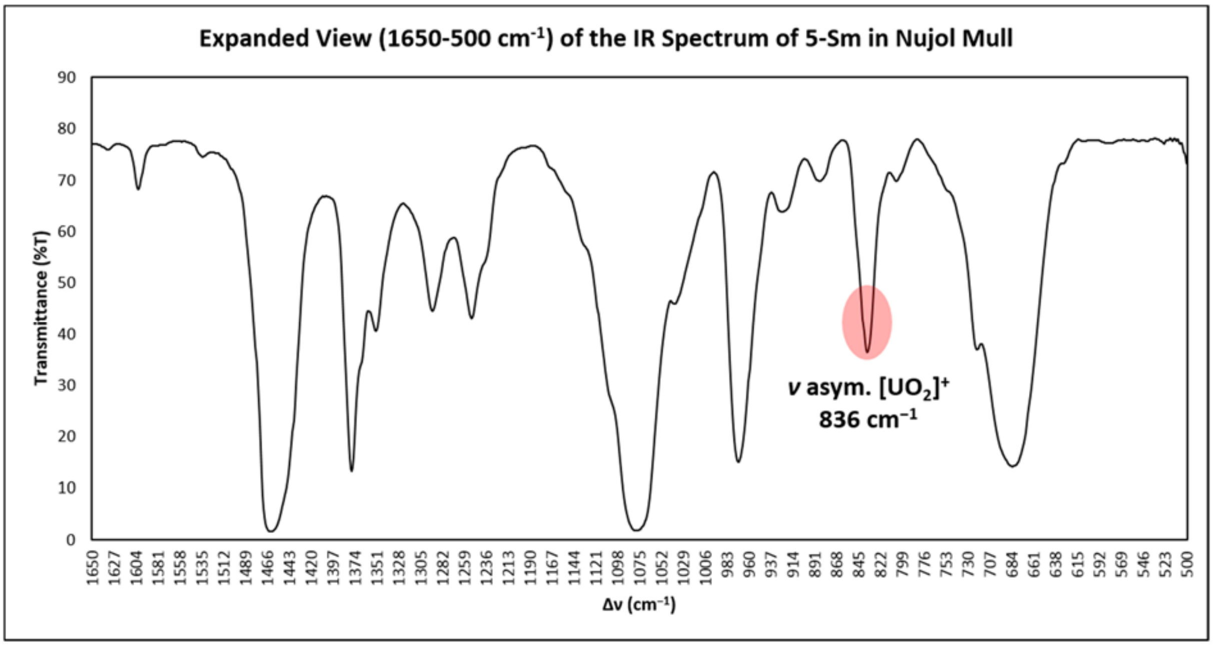

Figure S29. Expansion of the IR spectrum of **5-Sm** prepared via method *b* in nujol mull. Tentative assignment of v_{asymm} is labelled.

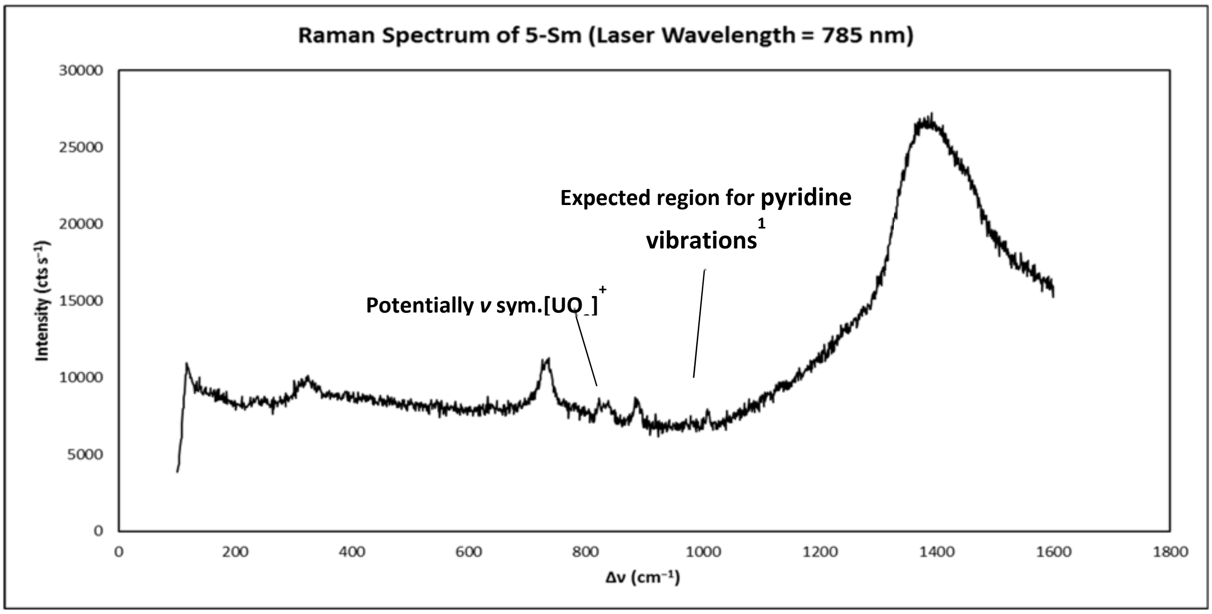

Figure S30. Raman Spectrum of **5-Sm**. Scanned from 1600-100 cm^{-1}, laser wavelength = 785 nm, laser power = 100%, laser focus = 100%, exposure = 25 seconds, scans = 15. Tentative assignment of v_{symm} is labelled.

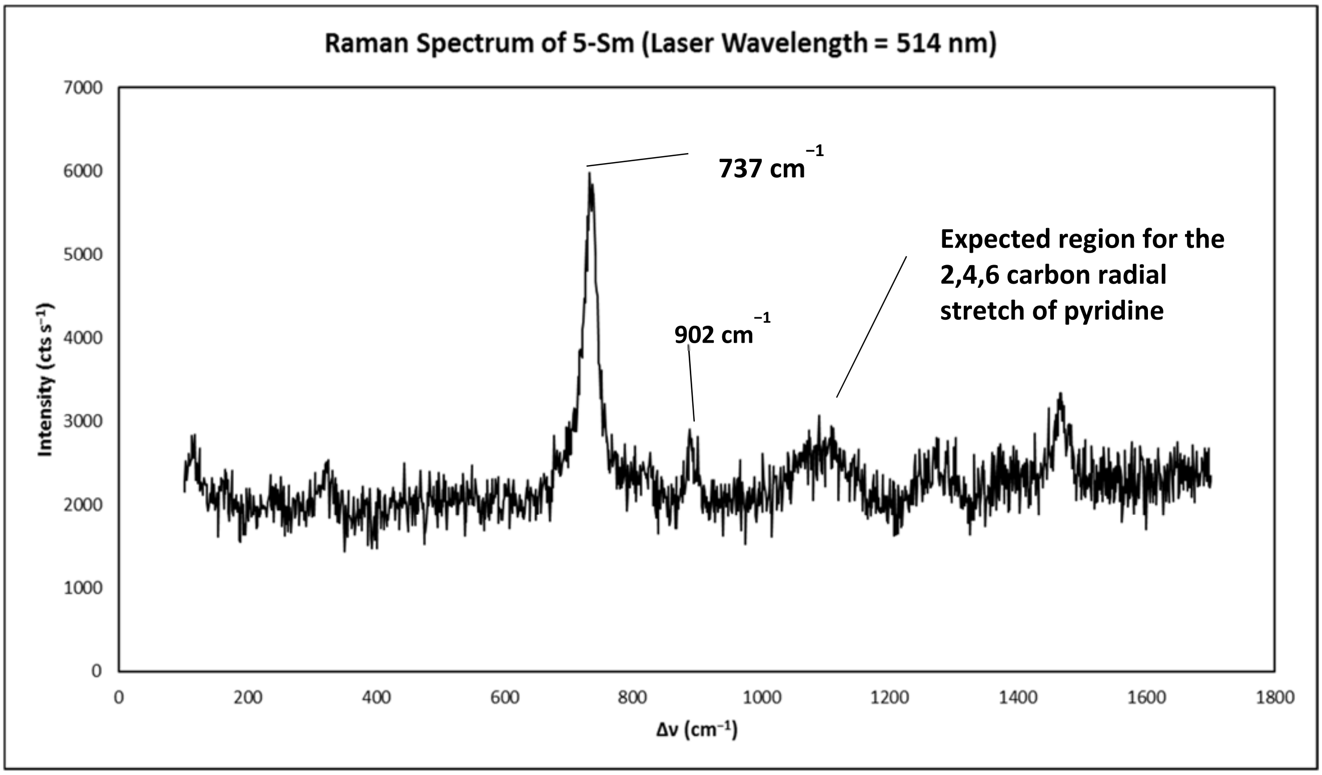

Figure S31. Raman Spectrum of **5-Sm**, with suggested UO_2 symmetric stretch labelled as 737 cm^{-1} and anticipated region for pyridine vibrations labelled, supporting the absence of coordinated pyridine. Scanned from 1700-100 cm^{-1}, laser wavelength = 514 nm, laser power = 50%, laser focus = 25%, exposure = 25 seconds, scans = 15.

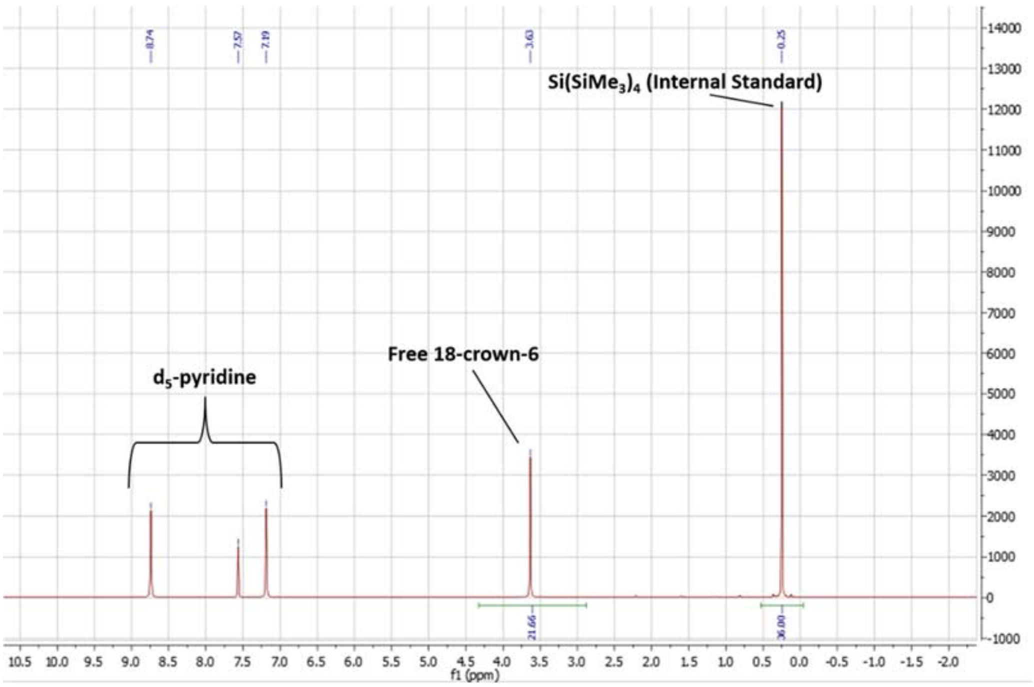

Figure S32. ^{1}H NMR spectrum of **5-Sm** in d_5-pyridine (500 MHz). $Si(Me_3)_4$ was added as an internal standard (in a 2:1 ratio relative to **5-Sm**) to quantify the amount of 18-crown-6 displaced from U upon solvation in pyridine. The integrals are approximately 22:36 for 18-crown-6:$Si(SiMe_3)_4$, in good agreement to the expected values (24:36).

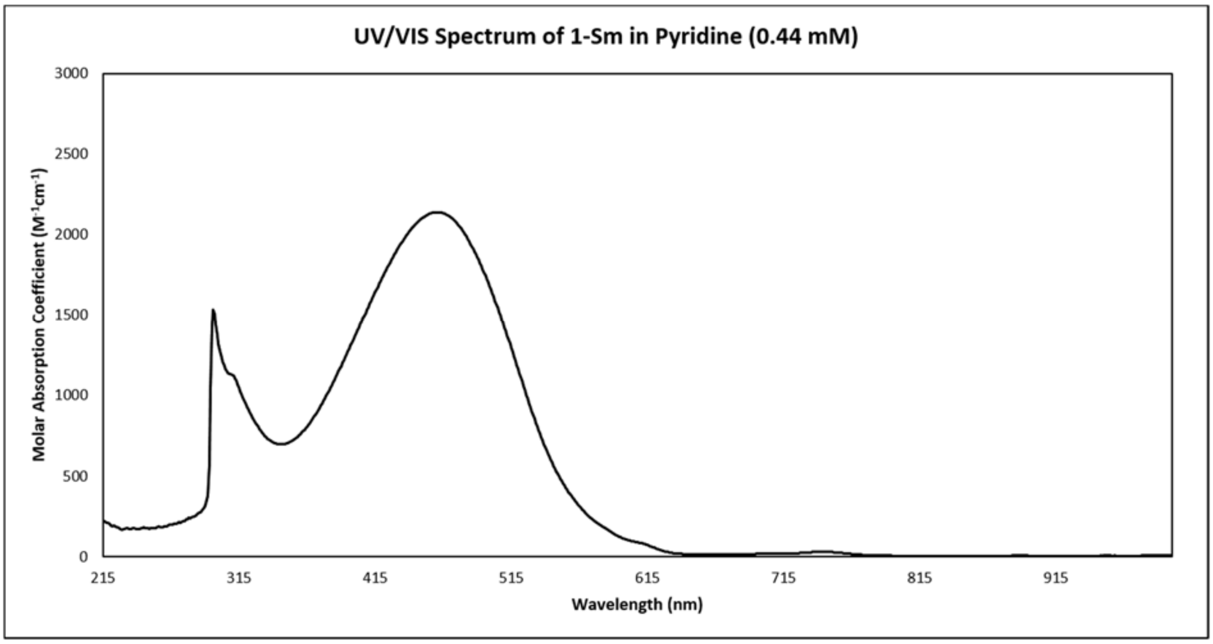

Figure S33. UV/VIS spectrum of **1-Sm** in pyridine.

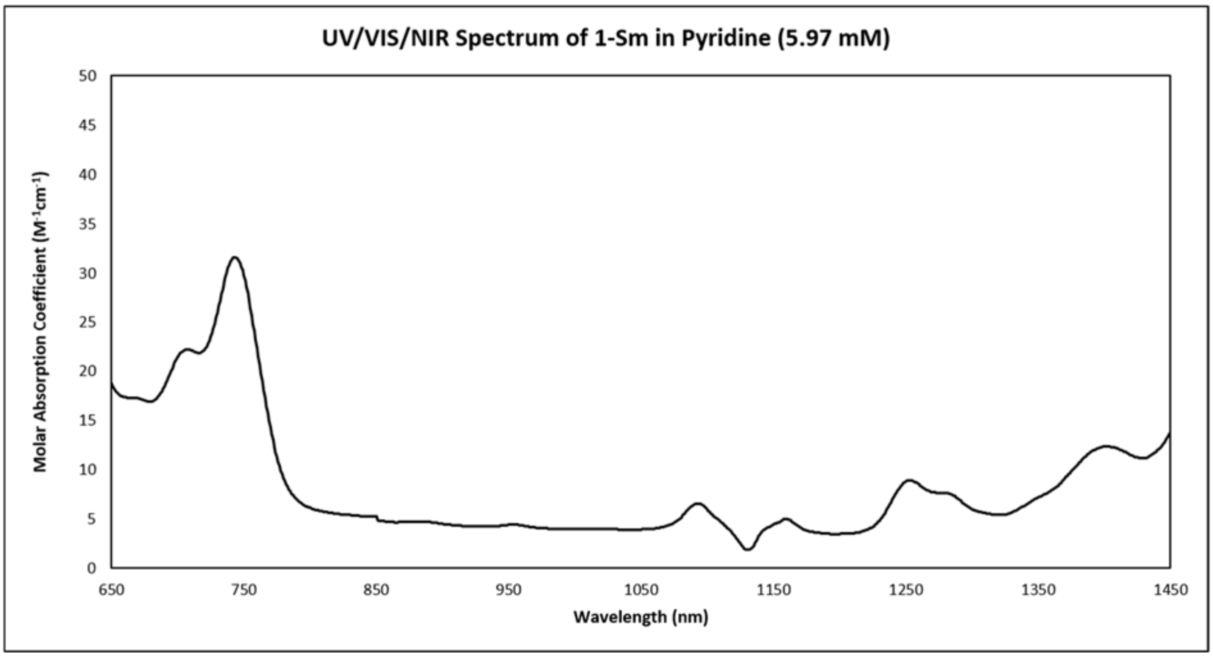

Figure S34. UV/VIS/NIR spectrum of **1-Sm** in pyridine.

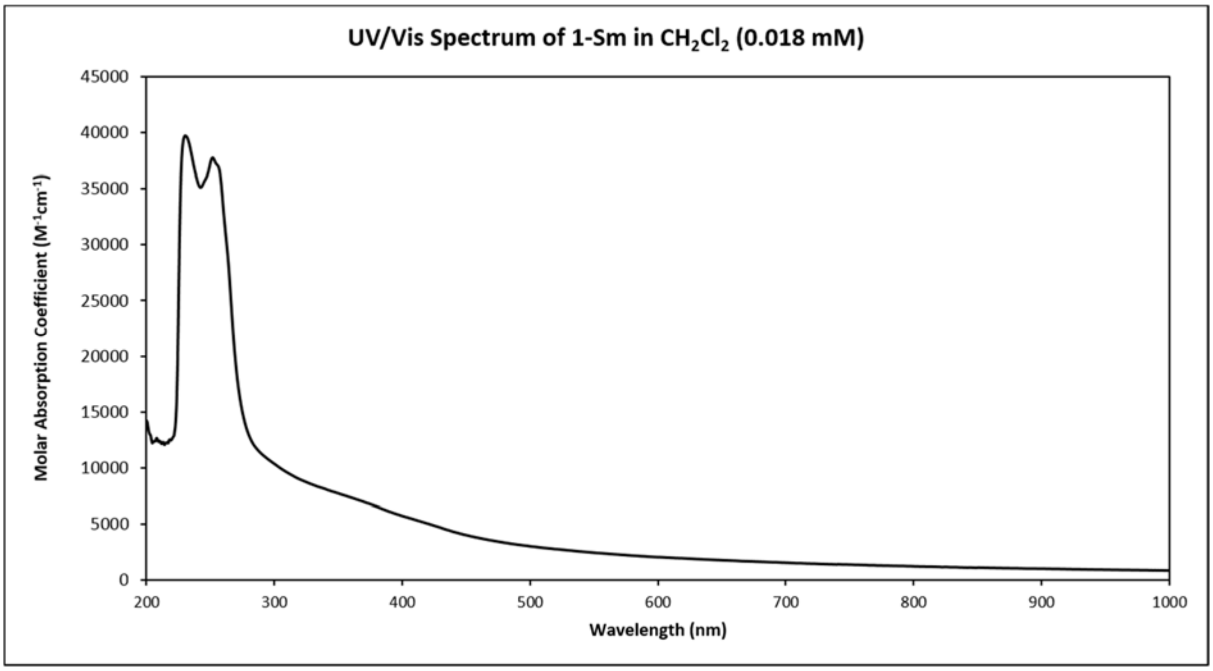

Figure S35. UV/VIS spectrum of **1-Sm** in CH_2Cl_2, dilute.

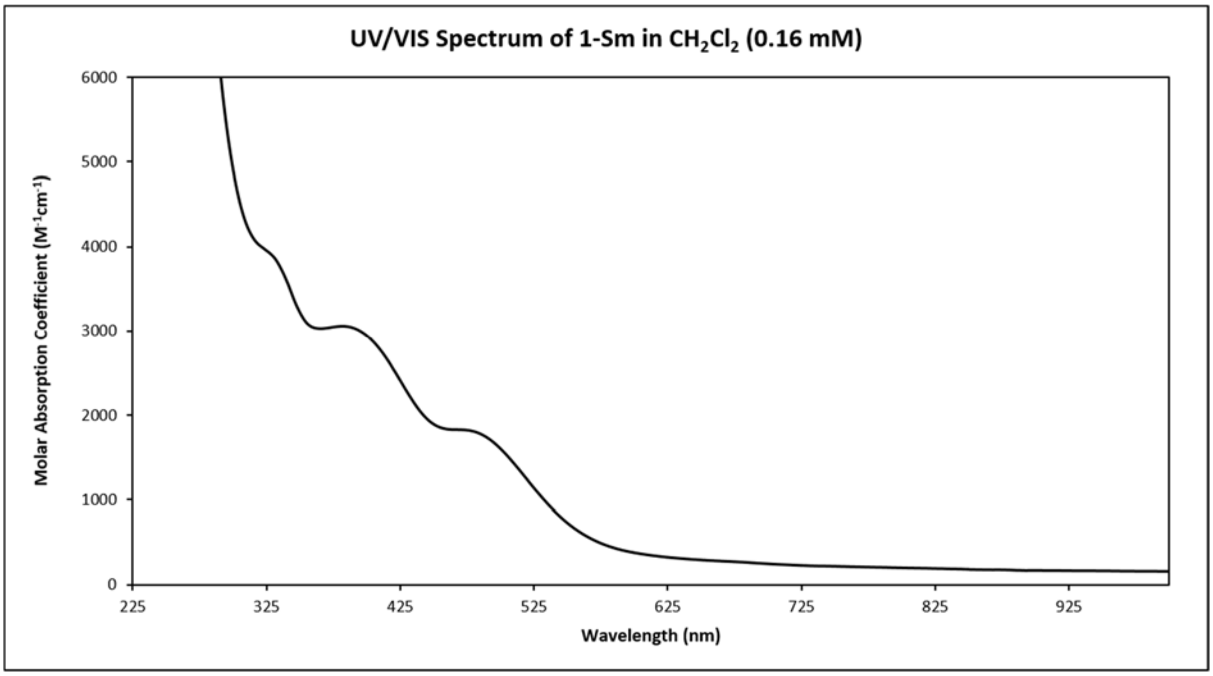

Figure S36. UV/VIS spectrum of **1-Sm** in CH_2Cl_2.

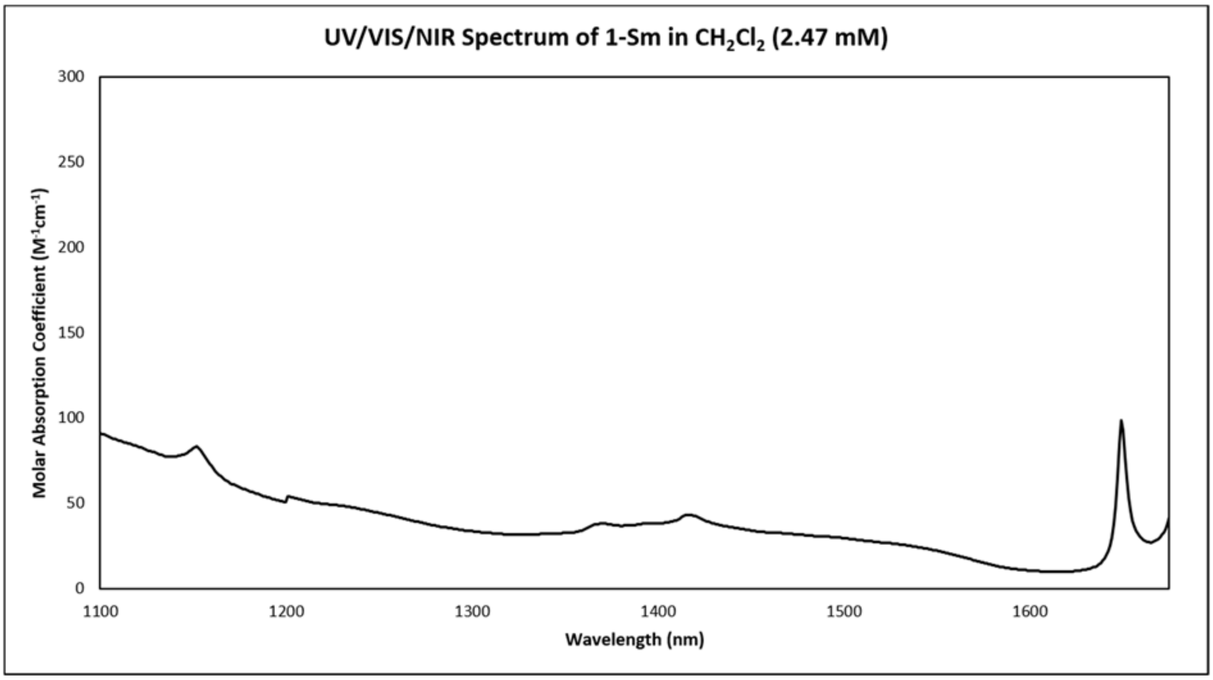

Figure S37. UV/VIS/NIR spectrum of **1-Sm** in CH_2Cl_2, concentrated.

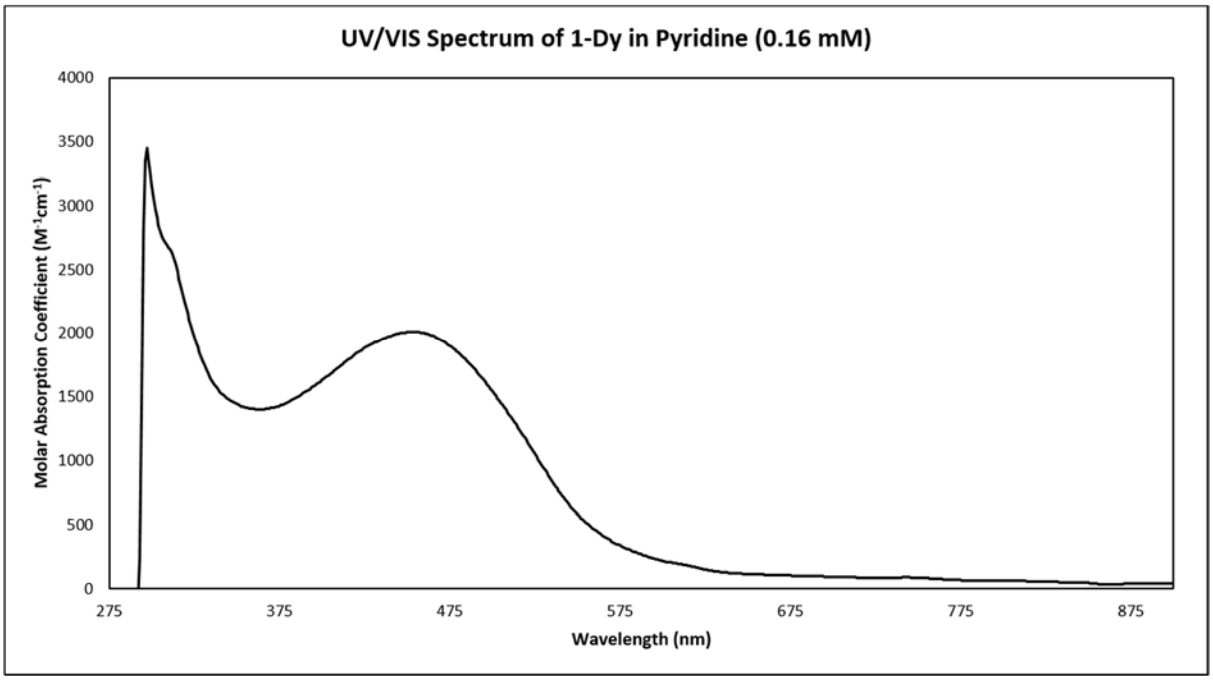

Figure S38. UV/VIS spectrum of **1-Dy** in pyridine.

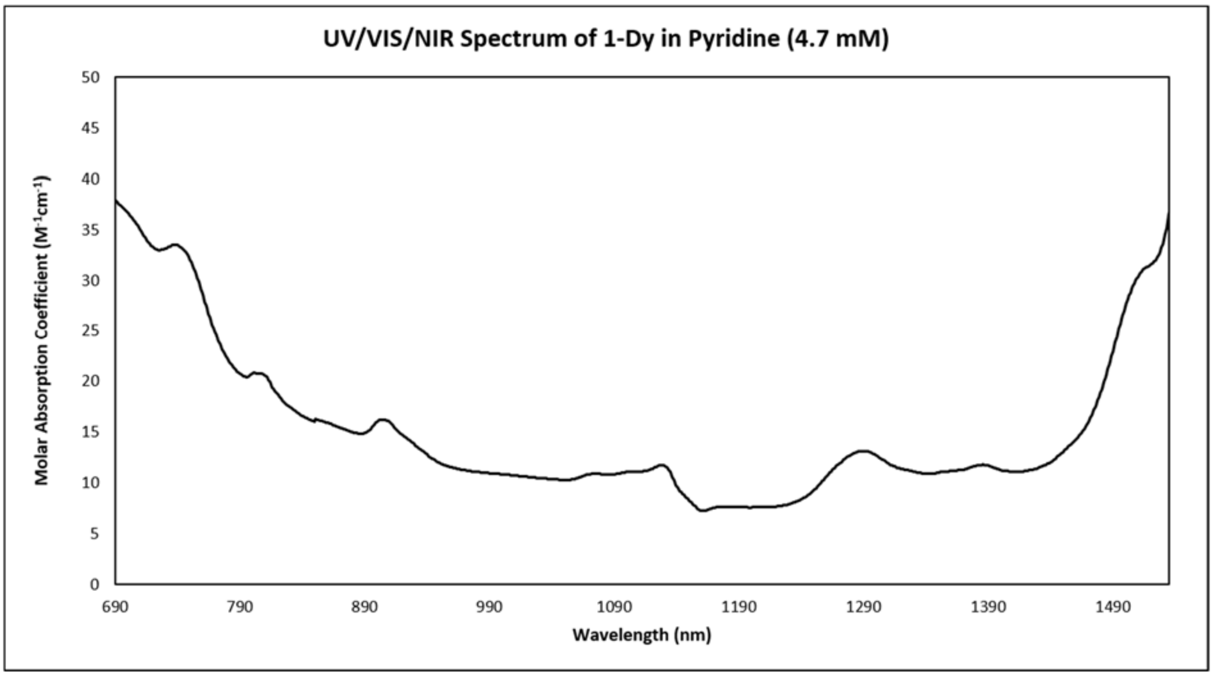

Figure S39. UV/VIS/NIR spectrum of **1-Dy** in pyridine, concentrated.

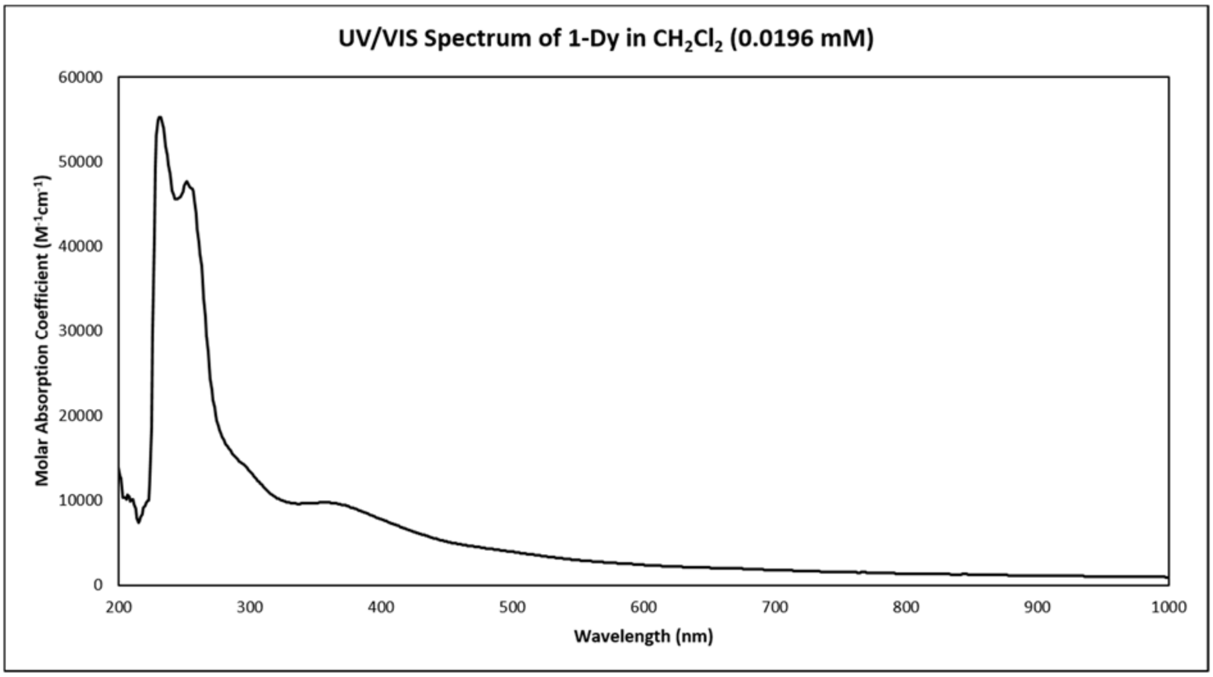

Figure S40. UV/VIS spectrum of **1-Dy** in CH_2Cl_2, dilute.

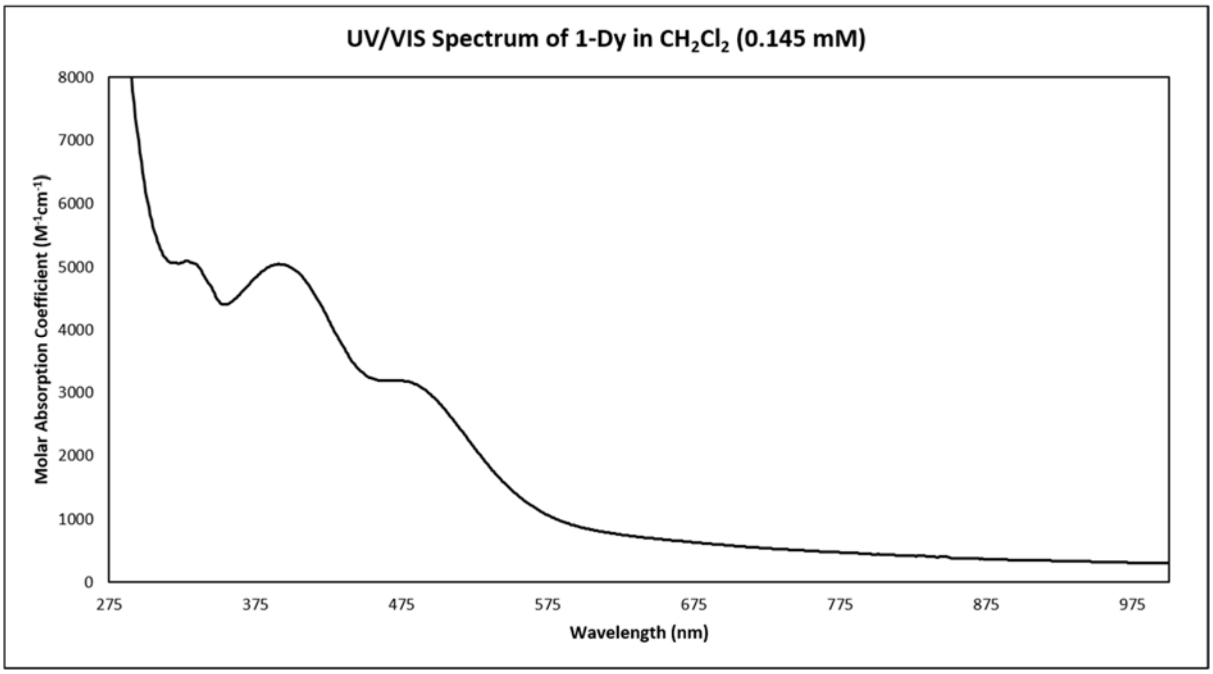

Figure S41. UV/VIS spectrum of **1-Dy** in CH_2Cl_2.

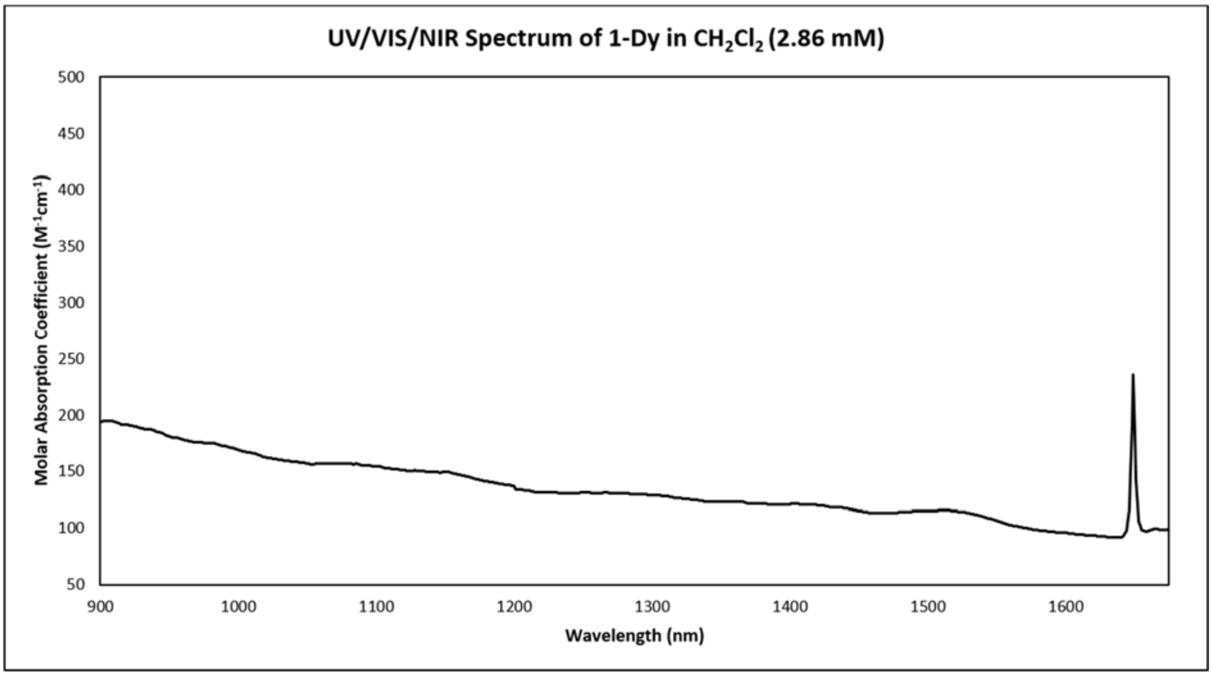

Figure S42. UV/VIS/NIR spectrum of **1-Dy** in CH_2Cl_2, concentrated.

References:

[1] M. P. Wilkerson, C. J. Burns, R. T. Paine, B. L. Scott, *Inorg. Chem.* **1999**, *38*, 4156.
[2] S. M. Mansell, B. F. Perandones, P. L. Arnold, *J. Organomet. Chem.* **2010**, *695*, 2814.
[3] D. M. Barnhart, C. J. Burns, N. N. Sauer, J. G. Watkin, *Inorg. Chem.* **1995**, *34*, 4079.
[4] I. L. Fedushkin, Y. A. Kurskii, V. I. Nevodchikov, M. N. Bochkarev, S. Müchle, H. Schumann, *Russ. Chem. Bull.* **2002**, *51*, 160.
[5] M. J. Monreal, R. K. Thomson, T. Cantat, N. E. Travia, B. L. Scott, J. L. Kiplinger, *Organometallics* **2011**, *30*, 2031.
[6] W. J. Evans, N. T. Allen, P. S. Workman, J. C. Meyer, *Inorg. Chem.* **2003**, *42*, 3097.
[7] K. Servaes, S. D. Houwer, C. Görller-Walrand, K. Binnemans, *Phys. Chem. Chem. Phys.* **2004**, *6*, 2946.
[8] G. M. Sheldrick, *Acta Cryst.* **2015**, *A71*, 3.
[9] G. M. Sheldrick, *Acta Cryst.* **2015**, *C71*, 3.
[10] O. V. Dolomanov, L. J. Bourhis, R. J. Gildea, J. A. Howard, H. Puschmann, *J. Appl. Crystallogr.* **2008**, *42*, 339.
[11] G. Sheldrick, *Acta Cryst.* **2008**, *A64*, 112.
[12] L. Farrugia, *J. Appl. Crystallogr.* **1999**, *32*, 837.
[13] P. Larkin, Infrared and Raman Spectroscopy: Principles and Spectral Interpretation; Elsevier: Oxford, UK, 2011; pp. 94-96
